# A combinatorial approach for the discovery of cytochrome P450 2D6 inhibitors from nature

**DOI:** 10.1038/s41598-017-08404-0

**Published:** 2017-08-14

**Authors:** Johannes Hochleitner, Muhammad Akram, Martina Ueberall, Rohan A. Davis, Birgit Waltenberger, Hermann Stuppner, Sonja Sturm, Florian Ueberall, Johanna M. Gostner, Daniela Schuster

**Affiliations:** 1 Division of Medical Biochemistry, Center for Chemistry and Biomedicine, Medical University of Innsbruck, Innsbruck, Austria; 2 Institute of Pharmacy/Pharmaceutical Chemistry, Computer Aided Molecular Design Group and Center for Molecular Biosciences Innsbruck (CMBI), University of Innsbruck, Innsbruck, Austria; 3 Griffith Institute for Drug Discovery, Griffith University, Brisbane, QLD 4111 Australia; 4 Institute of Pharmacy/Pharmacognosy and Center for Molecular Biosciences Innsbruck (CMBI), University of Innsbruck, Innsbruck, Austria

## Abstract

The human cytochrome P450 2D6 (CYP2D6) enzyme is part of phase-I metabolism and metabolizes at least 20% of all clinically relevant drugs. Therefore, it is an important target for drug-drug interaction (DDI) studies. High-throughput screening (HTS) assays are commonly used tools to examine DDI, but show certain drawbacks with regard to their applicability to natural products. We propose an *in silico* – *in vitro* workflow for the reliable identification of natural products with CYP2D6 inhibitory potential. In order to identify candidates from natural product-based databases that share similar structural features with established inhibitors, a pharmacophore model was applied. The virtual hits were tested for the inhibition of recombinant human CYP2D6 in a bioluminescence-based assay. By controlling for unspecific interferences of the test compounds with the detection reaction, the number of false positives were reduced. The success rate of the reported workflow was 76%, as most of the candidates identified in the *in silico* approach were able to inhibit CYP2D6 activity. In summary, the workflow presented here is a suitable and cost-efficient strategy for the discovery of new CYP2D6 inhibitors with natural product libraries.

## Introduction

The human cytochrome P450 2D6 (CYP2D6) enzyme is part of phase-I metabolism in which xenobiotics are oxidized to increase their excretion from the body^1^. Xenobiotics are chemicals that are foreign to the human body; examples include synthetic drugs, environmental chemicals, pesticides, herbicides, preservatives, flavourings and natural products, some of which are omnipresent in food and beverages^2^.

It is known that the mammalian CYP2D6 enzyme is one of the most polymorphic CYPs and metabolizes at least 20% of all clinically relevant drugs, such as those that act on the central nervous or cardiovascular system^1^. Due to the varying protein levels and metabolism rates of substrates, patients can be phenotypically classified as poor-, intermediate-, extensive- and ultra-metabolizers (PM, IM, EM, UM)^1^. Critical situations may occur if undiagnosed UM patients are treated with drugs, which are CYP2D6 substrates, because the accumulating metabolites may provoke serious side effects. In the case of the substrate codeine, UMs produce larger amounts of morphine than poor- or intermediate-metabolizers. The increased opiate concentration can lead to a depression of the respiratory tract and in the worst case scenario to death, as has been reported for paediatric patients^3^. In order to prevent such fatal drug-related side effects, the European Medicines Agency (EMA) has abandoned the use of codeine as an antitussive agent for children under the age of 12^4^. Therefore, it is of utmost importance to get comprehensive information about the metabolic profile of all ingested xenobiotics, especially of bioactive compounds such as drugs and natural products.

Both computer-based activity prediction studies^5–7^ and high-throughput screening (HTS) assays are commonly used tools to examine drug-drug interactions (DDI) and enzymatic activity of CYP-isoforms^8^. In general, the read-out of a CYP reaction is a fluorogenic or luminogenic signal^9^, depending on the probe-substrate. Such assay systems have also been used in investigations with herbal medicinal products^10^. With the increasing application of HTS assays in this specific research area, it has become evident that fluorescence-based assays are vulnerable to natural products, as these often exhibit intrinsic fluorescence or quenching. These effects can lead to a masking of enzyme inhibition or a simulation thereof, respectively^10^. For this reason, second-generation bioluminescence-based assays were developed, which exhibit greater versatility and sensitivity^9^.

**Figure 1:**
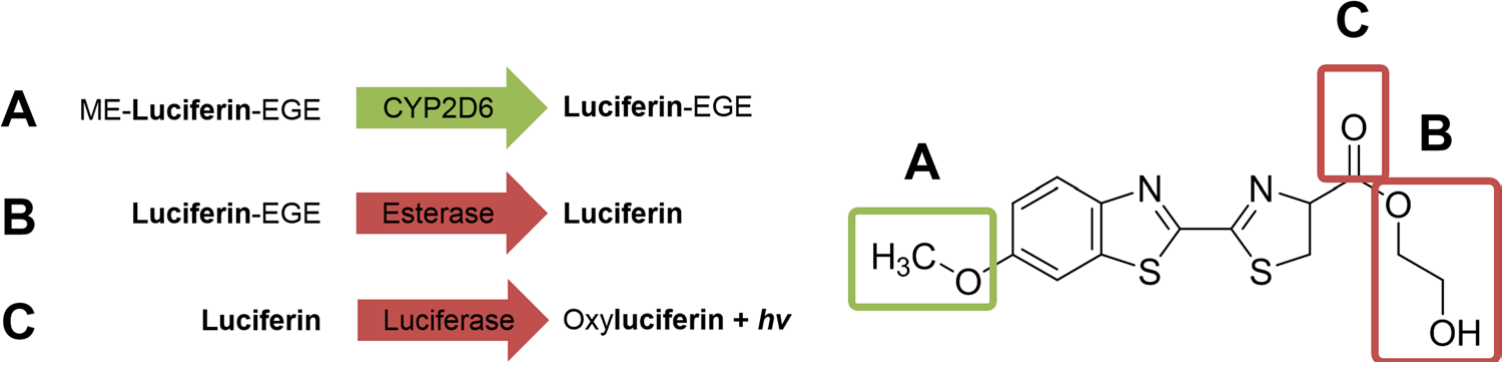
Essential steps of the luminescence-based, high-throughput P450-Glo CYP2D6 inhibition assay. (**A**) Methoxy-luciferin-ethylene glycol ester is a CYP2D6 substrate that is demethylated to luciferin-ethylene glycol ester in the presence of NADPH, which serves as an electron source. (**B**) The read-out of the CYP2D6 reaction is based on the treatment of the reaction mixture with the detection reagent that consists of a detergent, an unspecific esterase and a modified firefly-luciferase. (**C**) The esterase continuously generates luciferin, which is oxidized by the firefly-luciferase and thereby a stable luminogenic signal is produced.

CYP2D6 can use methoxy-luciferin-ethylene glycol ester (ME-luciferin-EGE) as a substrate. ME-luciferin-EGE is a luciferin derivative, which is demethylated to luciferin ethylene glycol ester (luciferin-EGE) via CYP2D6. Of note, luciferin-EGE is not yet a luciferase substrate (Fig. 1A). In a separately initiated detection reaction, an unspecific esterase hydrolyses the ethylene glycol ester and releases luciferin, which is accessible for the luciferase and ensures a glow-like signal over time^8^ (Fig. 1B and C). Although considered as second-generation and more rugged^9^, the bioluminescence-based assays are not flawless. A major limitation is that the signal output capacity is crucially dependent on the presence of the co-factors ATP and Mg^2+^ and the proper function of the luciferase^8^. Luminescence quenching has been considered in former studies^9, 11^. Furthermore, the polyphenol resveratrol was reported to inhibit firefly-luciferase in the lower micromolar range and thus to interfere with such bioluminescence-based assays^11^.

**Figure 2:**
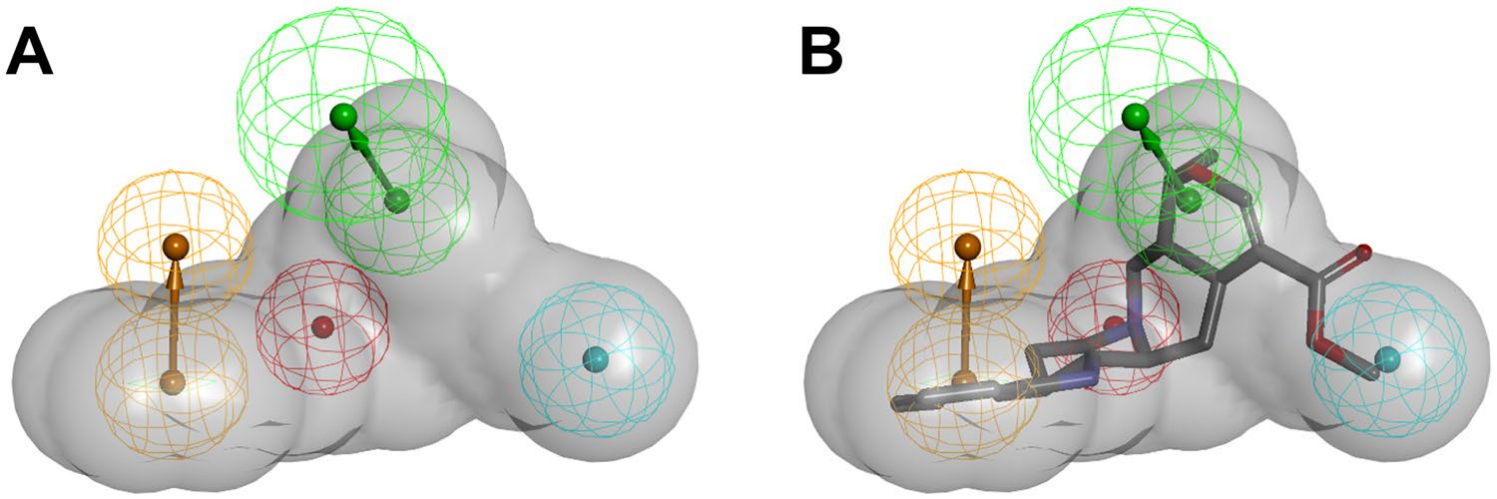
(**A**) The pharmacophore model consisting of typical physicochemical features for CYP2D6 inhibitors. Chemical features are color-coded: hydrophobic - cyan, hydrogen bond acceptor - green, positively ionisable group - red, aromatic ring - orange, shape and size restriction - grey. (**B**) The 3D structure of the known inhibitor ajmalicine is fitted into the model.

The aim of this study was to create a workflow for the discovery of potential CYP2D6 inhibitors in natural product libraries. We validated our approach with literature reported inhibition data and studied new, not yet analysed natural products for their enzyme inhibition potential. To maximize the success rate for the discovery of new inhibitors and to maintain low reagent costs, the compound selection was based on a virtual screening. For this, a previously reported CYP2D6 pharmacophore model^12^ was applied. A pharmacophore is the ensemble of steric and electronic features that is necessary to ensure the optimal supramolecular interactions with a specific biological target and to trigger (or block) its biological response^13^. This pharmacophore model was generated to find both natural products and synthetic drugs that share the same molecular properties as established inhibitors (Fig. 2).

The virtual hits with missing CYP2D6 inhibition data were selected for further testing using a bioluminescence-based P450-Glo CYP2D6 inhibition assay. A control step for the detection reaction helped to sort out false positives due to luminescence quenching. In order to visualize the possible binding poses of the novel CYP2D6 inhibitors in the active site cavity, we established a docking workflow based on the crystal structure 4WNT of CYP2D6 co-crystallized with the inhibitor ajmalicine^14^.

## Results

### Preparation of the *in silico* pre-selection workflow

#### Selection of the test-dataset

**Figure 3:**
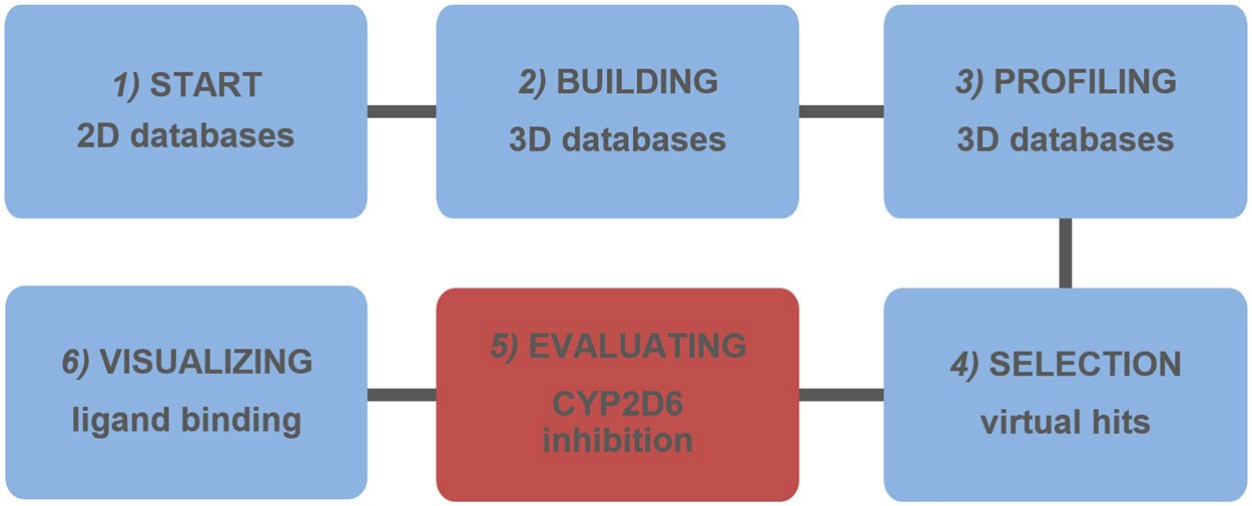
Workflow for the identification of novel CYP2D6 inhibitors in the 2D- natural products database: The starting points for the combinatorial workflow of *in silico* (blue) and *in vitro* (red) methods were 2D-databases that consisted of the chemical structure of natural products (1). Discovery Studio software was used to create (2) and virtually screen the 3D-databases for potential CYP2D6 inhibitors *in silico* (3). We selected those candidates for which no CYP2D6 inhibition data were available according to literature research (4). To investigate the inhibitory effect of the prospective inhibitors on CYP2D6 *in vitro*, a luminescence-based high-throughput assay was performed (5), including control reactions to avoid unspecific signals and thus false positive hits. In a last step, the confirmed inhibitors were docked *in silico* in the active site cavity to predict the binding pose and to estimate their ability to act as competitive inhibitors (6).

The starting point for the combinatorial approach to identify CYP2D6 inhibitors was the validation of the first two *in silico* steps of the workflow depicted in Fig. 3 that consisted of the building and virtual screening of a 3D-database, starting from a 2D-database. In search of suitable 2D-databases for the theoretical validation process, the PubChem Assay AID 891^15^ turned out to be a feasible dataset as it incorporates 1623 CYP2D6 inhibitors, 6338 non-inhibitors, and 1424 compounds with inconclusive behaviour towards CYP2D6. These compounds have been characterised previously with a bioluminescence-based CYP2D6 inhibition assay similar to the assay that we used for the CYP2D6 inhibition studies. The structure-data files (.sdf) for the active, inactive and inconclusive compounds were freely accessible at the PubChem website.

#### Building of a 3D-database

**Figure 4:**
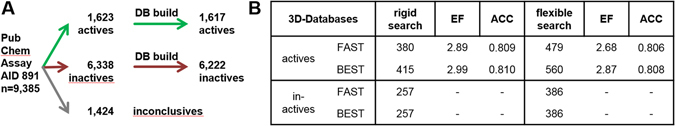
Validation of the workflow for building and profiling a 3D-database with the CYP2D6 inhibitor pharmacophore model. (**A**) The PubChem Assay AID891 was used as a test-database including 1623 active and 6338 inactive compounds. After the 3D conformer generation, 1617 actives and 6222 inactives remained in the database. (**B**) To generate the 3D-database we used the FAST and the BEST modes, respectively. For the profiling of the four 3D-databases, we applied a rigid and a flexible search with the already published pharmacophore model, respectively. The various settings yielded different hit lists, which compositions were quantified using the EF and ACC.

The building of a 3D-database was the first step of the validation process and was performed for the active and inactive compounds retrieved from the PubChem Assay AID 891. The maximum amount of energetically accessible conformations of each compound in the 3D-database is defined likewise the speed and quality with which it is built up, reflecting the FAST and the BEST mode^16^, respectively. We generated 3D-databases with a maximum of 255 conformations for the active (1623) and the inactive (6338) compounds from PubChem Assay AID 891, in the FAST and the BEST setting mode, respectively. The final multi-conformational 3D databases consisted of 1617 compounds in the active (FAST and BEST) and 6222 compounds in the inactive (FAST and BEST) 3D-databases, respectively (Fig. 4A).

#### Screening of a 3D-database with the pharmacophore model

After building the 3D-databases in the different software settings, we screened the four 3D-databases *i.e*. 1617 actives (FAST and BEST) and 6222 inactives (FAST and BEST) with our CYP2D6 inhibitor pharmacophore model^12^. We applied a rigid and a flexible search^16^ on the two active and the two inactive 3D-databases, respectively, and retrieved eight corresponding hit lists (Fig. 4B). Interestingly, the inactives database built in the FAST and BEST mode resulted in the same amount of hits independent from the screening algorithm (Fig. 4B).

#### Deciding on a workflow based on theoretical quality metrics

After the virtual screening of the 3D-databases, we had to figure out the workflow that provides the most active hits in relation to the inactive hits in our test-database by calculating the enrichment factor (EF)^17^. With the EF we were able to calculate the enrichment of active compounds in the virtual hit list compared to a random selection of compounds^18^. We found that building a 3D-database for the active compounds in the FAST mode and applying a rigid and a flexible search resulted in an EF of 2.89 and 2.68, respectively, whereas building the 3D-database for the active compounds in the BEST mode yielded corresponding EF-values of 2.99 and 2.87 (Fig. 4B). For assessing the predictive power of an antitarget screen, also the correct classification of inactive compounds is crucial. Therefore, the power of the screening settings to discriminate active and inactive inhibitors was calculated as the respective accuracies (ACC) (Fig. 4B). According to these results, we decided to generate the 3D-database for the prospective identification of CYP2D6 inhibitors in the BEST mode and apply a rigid search for the *in silico* screening.

### Application of the validated pre-selection workflow on natural product databases

We applied the determined *in silico* workflow on 17 in-house databases that contained 2147 single substances mainly derived from plants (Supplementary Table S1). The 17 2D-databases were provided by four different university groups and one was downloaded from the SPECS-homepage, a commercial provider of compounds that also offers databases consisting of natural products. The detailed results of the generation and the virtual screening of these 3D-databases can be found in the supplementary data section (Supplementary Table S1).

### Selection of the *in silico* hits for *in vitro* testing

**Figure 5:**
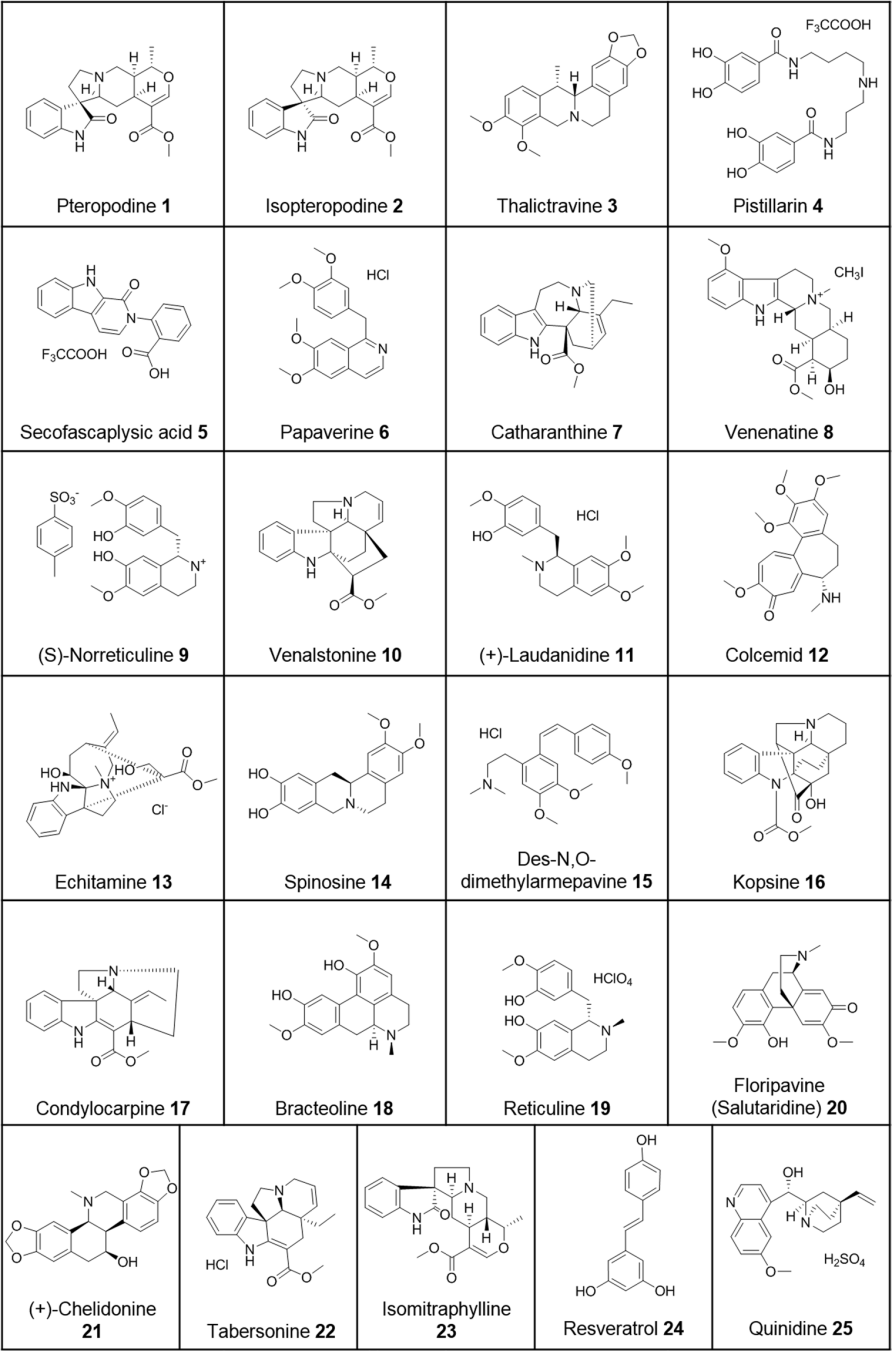
2D-Structures of the compounds tested *in vitro*.

The screening of the 3D-databases resulted in overall 75 unique hits that fitted the pharmacophore model and therefore were supposed to be CYP2D6 inhibitors. In order to separate synthetic, partially modified, and natural compounds and to find out if there are already known CYP2D6 inhibitors in the hit list, we performed a literature research using the SciFinder database^19^. The activity values of the non-selected hits are available as supporting information (Supplementary Table S2). We were able to identify 23 unmodified natural compounds for which no IC_50_ data on CYP2D6 was available (**1**–**23**, Fig. 5). For one compound, papaverine (**6**), we found two citations which suggest a possible CYP2D6 inhibition^20, 21^ and for catharanthine (**7**) we found a source reporting an inhibitory effect but no IC_50_ value^22^. One compound, salutaridine (**20**), is known to be a product of CYP2D6 after phenol-coupling of reticuline (**19**) and thus is a precursor for endogenous morphine biosynthesis^23^. So, it was interesting to investigate, if these three compounds exhibit an inhibitory effect on CYP2D6. In addition to the 23 selected hits from the pharmacophore screening, we tested the polyphenol stilbene derivative resveratrol (**24**). This compound differs in its chemical composition from the virtual hits and does not fit into the pharmacophore model. Furthermore, **24** shows a multitude of effects on different targets and is therefore called an invalid metabolic panacea (IMP)^24^. We used **24** as negative control for the *in vitro* assay. As a positive control, we chose quinidine (**25**), a commonly used inhibitor of CYP2D6^25^. A list of all compound names can be found in the supplementary data section (Supplementary Table S3) and the 2D-structures are depicted in Fig. 5.

### Evaluation of the CYP2D6 inhibition *in vitro*

#### Pre-screen at a concentration of 100 µM

We tested 23 natural compounds that fitted the pharmacophore model for CYP2D6 inhibitors and resveratrol, a known firefly luciferase inhibitor^11^, in the P450-Glo CYP2D6 inhibition assay (Fig. 1). Their CYP2D6 inhibitory potential was determined at a concentration of 100 µM in a final reaction volume of 50 µl. For the assessment of the biological activity, a concentration of 100 µM is frequently used to decide on the classification of a compound as completely inactive^26, 27^. All compounds were dissolved in DMSO and the concentration thereof in the final reaction volume was 1%, according to the limitations indicated in the manufacturer’s protocol. Compound **25** is a standard inhibitor of CYP2D6^25^, and was used at a final concentration of 1 µM as the positive control, whereas 1% DMSO was used as negative control. To take into account the background luminescence, which may derive from a non-enzymatic conversion of ME-luciferin-EGE to luciferin, we included a ‘minus P450’ control that contained no CYP2D6 enzyme. In order to quantify CYP2D6 activity, we subtracted background values prior to calculating the percentage of total luminescence (% TL) obtained from the solvent control (set to 100%). Low % TL indicates low CYP2D6 activity.

**Figure 6:**
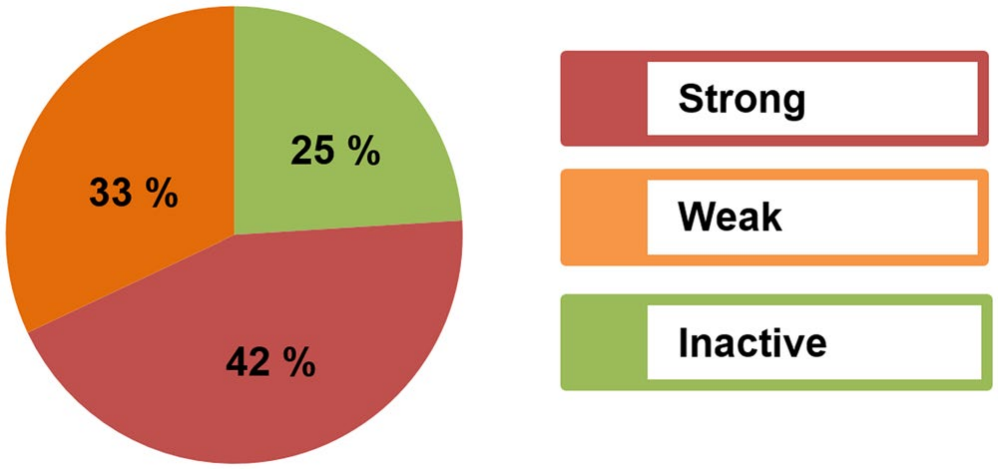
Screening for CYP2D6 inhibitory properties of the preselected compounds at a concentration of 100 µM. A strong or intermediate inhibition was observed for 18 out of 24 compounds. Only 6 compounds exhibited more than 50% of total luminescence, indicating inactivity at 100 µM.

We found that 10 compounds (*i.e*. 42%) exhibited a strong, and 8 compounds (*i.e*. 33%) a weak inhibition of CYP2D6, of 0–10 and 10–50% TL at 100 µM, respectively. Only 6 compounds (*i.e*. 25%) proved to be completely inactive and displayed more than 50% TL at 100 µM (Fig. 6). The strongest inhibitor of CYP2D6 in our natural products based database was chelidonine (**21**) exhibiting 0.45% TL at 100 µM, which reflects a CYP2D6 inhibition of 99.55% at the given concentration. Isopteropodine (**2**) was the weakest inhibitor of the tested compounds with 43.22% TL at 100 µM (CYP2D6 inhibition of 56.78%).

#### Detection control

The commercially available P450-Glo CYP2D6 inhibition assay is based on the demethylation of the ME-luciferin-EGE to luciferin-EGE by CYP2D6 enzyme. The quantification of the CYP2D6 reaction is possible due to the treatment of the reaction mixture with the P450-detection-reagent after exhaustive CYP2D6 reaction. This detection-reagent consists of *i)* a detergent that stops the CYP2D6 enzyme reaction, *ii)* an unspecific esterase that continuously releases luciferin and *iii)* a modified firefly-luciferase, thus a stable luminogenic signal is generated, which can be quantified by a luminometer (Fig. 1). The detection reaction is incorporated in the P450-Glo CYP2D6 inhibition assay. However, we implemented additional controls for unspecific interactions of the test compounds with the detection reaction. For this purpose, the previously established luminescence quenching control reaction reported by Modarai *et al*.^9^ was adapted to our requirements with modifications for the use of larger sample volumes. In addition, Modarai *et al*. converted ME-luciferin-EGE to luciferin in the absence of a test compound and then evaluated the inhibition of the luciferase reaction.We conducted the control step by using pure luciferin-EGE, esterase, luciferase and a test compound in the absence of CYP2D6 (Supplementary Table S4). A concentration of 0.75 µM luciferin-EGE was selected by referring to the turnover number (kcat) of ME-luciferin-EGE of 7.1 min^−1^ reported by Cali *et al*.^28^.

For this detection reaction control, we set the concentration of the 23 compounds and resveratrol at 50 µM in the final reaction volume of 100 µl, in order to reflect the same final concentration that is achieved in the P450-Glo CYP2D6 inhibition assay after addition of the detection reagents (Supplementary Table S5). The quantification of the P450-Glo detection reaction was performed by subtracting the background values prior to calculating the percentage of total luminescence (% TL) based on the total luminescence obtained from the solvent control (set to 100%).

**Figure 7:**
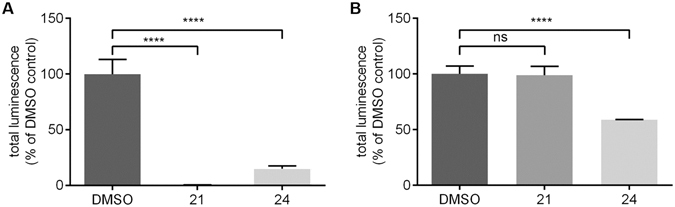
Inhibitory properties of **21** and **24** in the CYP2D6 inhibition assay and in its detection reaction. (**A**) Data from the CYP2D6 inhibition assay. (**B**) Data from the detection reaction. Both compounds **21** and **24** showed a strong and an intermediate inhibitory potential of CYP2D6, respectively, in graph (**A**), whereby only resveratrol (**24)** inhibited the detection reaction to a considerable amount as can be seen in graph (**B**). As the detection reaction is part of the P450-Glo CYP2D6 inhibition assay, it is necessary to ensure that the compounds do not interfere with this reaction. Shown is the mean with range of triplicate measurements of two different experimental days.

We found that the IMP resveratrol (**24**), showed 58.85% TL at a concentration of 50 µM reflecting a highly significant inhibition of 41.15% of the detection reaction. Chelidonine (**21**) in contrast, a compound that fits the pharmacophore model, was the most active compound in our dataset. It exhibited 98.77% TL at 50 µM, therefore inhibiting the detection reaction to a negligible and non-significant amount of 1.23% (Fig. 7).

#### IC_50_ determination of active and intermediate compounds

We examined the active (0–10% TL) and weakly active (10–50% TL) compounds from the pre-screening at 100 µM on the P450-Glo CYP2D6 inhibition assay, in a concentration-dependent manner.

Thirteen compounds exhibited an IC_50_ value of lower than 10 µM. The strongest inhibitors of the CYP2D6 enzyme were compounds **21**, **19** and **9**, as they showed IC_50_ values of 22.45, 54.70 and 63.64 nM. The IC_50_ values of the remaining ten compounds *i.e*. **14**, **15**, **11**, **22, 7** and **18**, **8**, **3**, **6**, **17** were distributed equally in the range of 0.1–1 and 1–10 µM, respectively.

For the calculation of the IC_50_ values, we included all available six data-points of the respective concentration-response curves. Only **18** exhibited a non-monotonic response at low-level concentrations and therefore two of six available concentration points were excluded^29^.

The respective IC_50_ values and 95% confidence intervals are reported in Table 1 and the inhibition curves of the three most potent inhibitors can be found in the Supplementary data (Supplementary Fig. S1).

**Table 1:** Comparison of the CYP2D6-inhibition to the detection reaction-inhibitory potential of the tested natural compounds.

	Compound	P450-Glo CYP2D6 Inhibition Assay			P450-Glo Detection Reaction			
		IC_50_ [nM]	95% CI [nM]	number of tested concentrations	MEAN	DMSO 1%	MEAN Diff.	p < 0.05*
**actives**	**21**	22.45	11.92–42.28	6	98.77	100	1.23	No
	**19**	54.70	26.34–113.6	6	98.94	100	1.06	No
	**9**	63.64	25.23–160.5	6	92.36	100	7.64	Yes
	**14**	116.9	64.44–212.1	6	95.27	100	4.73	Yes
	**15**	197.8	11.17–3503	6	93.77	100	6.23	Yes
	**11**	380.1	21.25–6799	6	94.14	100	5.86	Yes
	**22**	418.6	14.01–12502	6	104.20	100	−4.24	Yes
	**7**	582.2	61.11–5547	6	93.59	100	6.41	Yes
	**18**	2109	767.0–5801	4	87.70	100	12.30	Yes
	**8**	4601	4222–5015	6	90.86	100	9.14	Yes
	**3**	5137	1178–22406	6	91.77	100	8.23	Yes
	**6**	7022	5401–9129	6	82.48	100	17.52	Yes
	**17**	8157	6785–9806	6	92.66	100	7.34	Yes
**weak actives**	**24**	17367	6964–43309	6	58.85	100	41.15	Yes
	**20**	23991	9111–63173	6	97.06	100	2.94	No
	**1**	33816	18780–60889	6	92.50	100	7.50	Yes
	**5**	50353	23998–105652	6	79.54	100	20.46	Yes
	**2**	89093	70075–113273	6	92.43	100	7.57	Yes
**inactives**	**4**				86.39	100	13.61	Yes
	**10**				96.34	100	3.66	No
	**12**				91.99	100	8.01	Yes
	**13**				92.54	100	7.46	Yes
	**16**				89.70	100	10.30	Yes
	**23**				99.41	100	0.59	No

The compounds are ranked according to their corresponding IC_50_ values for CYP2D6 inhibition. *The p value refers to the ability of the respective compound to inhibit the P450-Glo detection reaction significantly.

### Preparation of an *in silico* docking workflow for CYP2D6 inhibitors

The ligand-based pharmacophore model successfully identified novel CYP2D6 inhibitors. Obviously, the chemical functionalities incorporated in the model (aromatic interaction, positively ionizable nitrogen, hydrogen bond acceptor and hydrophobic group) were common to well-known and newly identified inhibitors. However, the chemical features of ligand-based models do not necessarily directly correspond to specific protein-ligand interactions. Additionally, the well-known flexibility of the CYP2D6^5^ was only partially accounted for by the size of the model features (diameter of 2.6 Å). To address the question of whether the most potent, newly identified inhibitors could also form the same molecular interactions with CYP2D6 as the known ligands, docking studies were conducted.

The crystal structure of CYP2D6 has been solved^30^. Also further complexes of CYP2D6, co-crystallized with inhibitors, are accessible. Currently, the Protein Data Bank^31^ (PDB, www.rcsb.org) contains ten crystal structures of CYP2D6, whereas one structure, *i.e*. 2F9Q^30^, represents the ligand-free crystal structure and nine, *i.e*. 3QM4^32^, 3TBG^14^, 3TDA^14^, 4WNT^14^, 4WNU^14^, 4WNV^14^, 4WNW^14^,4XRY^33^ and 4XRZ^33^, exhibit the crystal structure co-crystallized with at least one ligand. Our main research interest was the visualization of CYP2D6 inhibitors in the active site cavity. Therefore, we focused on developing a docking workflow based on the crystal structure of CYP2D6, co-crystallized with an inhibitor.

We found 4WNU^14^, 4WNT^14^ and 4XRZ^33^ as the most suitable PDB entries for our research effort. The co-crystallized ligands for 4WNU and 4WNT are natural products *i.e*. quinidine and ajmalicine^14^. Contrasting them, 4XRZ comprises the CYP2D6 metabolite of a synthetic ß-secretase inhibitor^33^. Regarding the results from the validation re-docking process that can be found in the Supplementary data (Supplementary Fig. S2), 4WNT was used for the docking of the inhibitors in the active site cavity.

### Visualization of the ligand binding using the *in silico* docking model

For the prediction of the protein-ligand interactions, we docked the three strongest inhibitors *i.e*. **21**, **19** and **9**, into the active site cavity of CYP2D6. Based on the crystallographic data, Phe120, Glu216 and Asp301 were identified as important amino acid residues in the active site cavity for ligand interaction. It was found previously that Phe120 orientates ligands with respect to the heme by π-π stacking after they have been attracted by Glu216 and/or Asp301 via ionic interactions^14, 30^. Chelidonine (**21**), reticuline (**19**) and (*S*)-norreticuline (**9**) exhibited an IC_50_ value of 22.45, 54.70 and 63.64 nM, respectively and were the strongest inhibitors of our dataset *in vitro*.

All three inhibitors could be docked into the active site cavity of CYP2D6 and displayed an interaction with Phe120. This interaction was formed between the aromatic substructure of the inhibitor molecule and the benzene ring of the amino acid located on the B’-C loop. The compounds **21**, **19** and **9** were placed at distances of 3.1, 3.6 and 3.8 Å to Phe120, respectively. Ionic interactions between Glu216 located on the F’-helix and the protonated nitrogen from the inhibitors could be found in the best docking solution of **21**, **19** and **9** with an approximate distance of 3.6, 3.1 and 5.1 Å, respectively. **21** and **19** formed hydrogen bonds with Asp301 at a distance of 4.4 and 3.3 Å respectively. The protonated nitrogen of **9** was accessible for Asp301 located on the I-helix with a distance of 3.3 Å. A list of the observed protein-ligand interactions and their respective distances can be found in the Supplementary data section (Supplementary Table S6).

**Figure 8:**
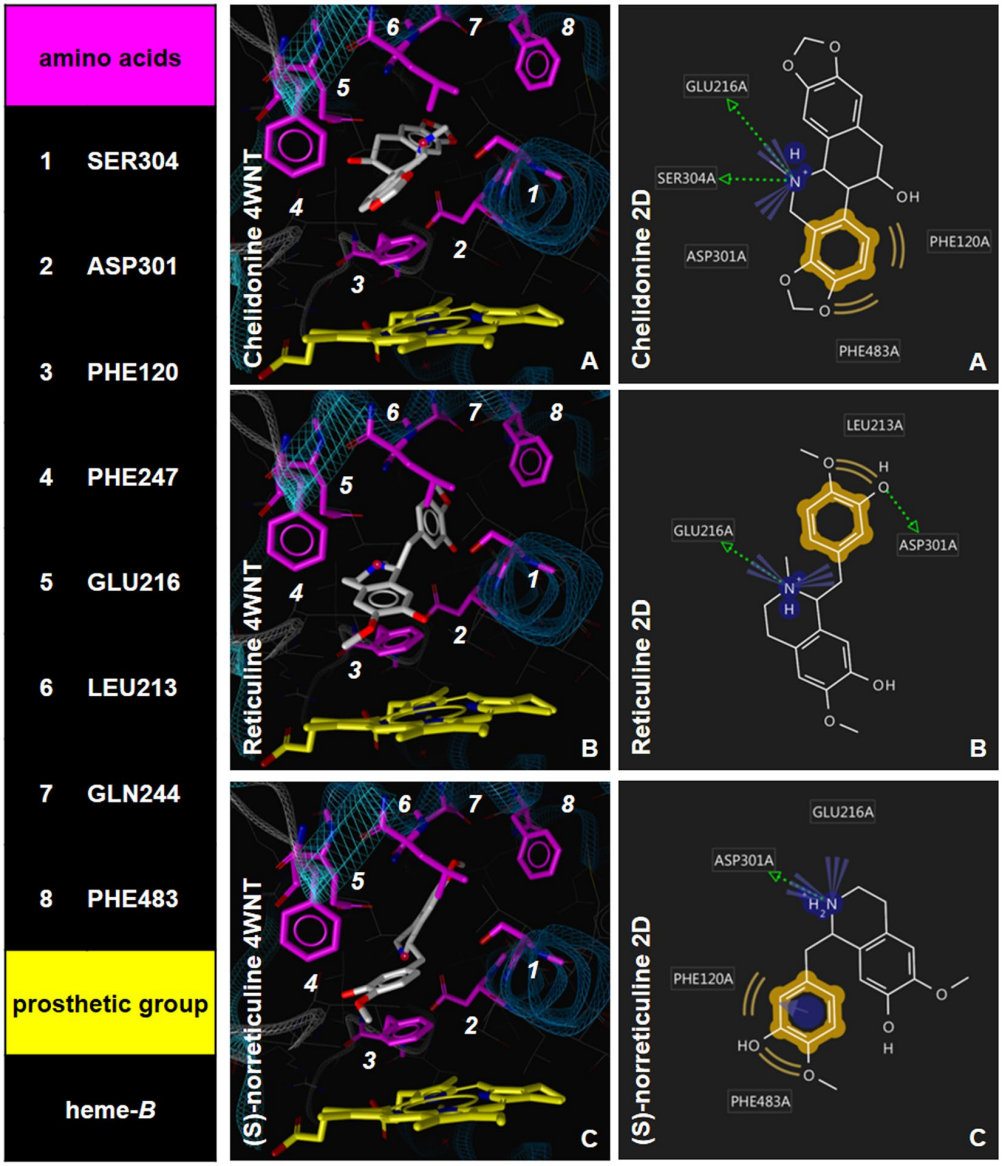
Visualization of the nanomolar CYP2D6 inhibitors **21**, **19** and **9** in the active site cavity (**A**), (**C**) and (**E**). The amino acids highlighted in magenta were found as important interaction and binding partners in previous studies. Heme-b as the prosthetic group is highlighted in yellow and coordinates an iron atom in the middle of the molecule that enables the oxidative metabolization of substrates. (**B**), (**D**) and (**F**) illustrate the 2D structures of the docked inhibitors and the respective interactions that are formed between the ligands and amino acids of the active site cavity of CYP2D6. Protein-ligand interactions are color-coded: green arrow - hydrogen bond donor, blue star - positively ionizable group, yellow - hydrophobic contact, blue circle with arrow - aromatic interaction.

The most prominent interactions of the docked ligands with the amino acids of the active site cavity are depicted in Fig. 8. In accordance with the *in vitro* data, the newly identified CYP2D6 inhibitors are predicted to form essential protein-ligand interactions that are observed in X-ray structures of other CYP2D6-inhibitor complexes, most of them also corresponding to the chemical features from the pharmacophore model. A more comprehensive analysis of binding poses within several X-ray structures of CYP2D6 can be found in the Supplementary data (Supplementary Fig. S3 and Tables S7–9).

## Discussion

In this study, we present a robust workflow for the investigation of CYP2D6 inhibition by natural products. The implementation of an *in silico* pre-selection method of the compounds tested *in vitro* may help to save costs and to avoid unnecessary chemical waste. Besides the identification of previously unknown CYP2D6 inhibitors, our motivation was to highlight the possible limitations that may be encountered when structurally diverse compounds are evaluated in a high-throughput screening assay.

To obtain our targets, we virtually screened compound databases for possible CYP2D6 inhibitors with a pharmacophore model^9^. In the current study, this model has been tested for its predictive power for the first time and an excellent performance could be demonstrated. Focusing on the results from the pre-screen assay (Fig. 6), 18 compounds exhibited a strong or weak CYP2D6 inhibition at 100 µM, which indicated a true positive hit rate of 75%. For comparison, the high throughput screening of random compounds (PubChem Assay AID 891) had shown a success rate of approximately 20%. Thus, the here reported workflow represents as a reliable strategy to enrich CYP2D6 inhibitors in a hit list when screening large databases. However, when applied to larger databases, the performance of the model may vary. In the PubChem dataset, the pharmacophore model correctly predicted, depending on the screening settings, up to 34.6% active and 93.8% inactive compounds, respectively (Fig. 4B). In the prospective screening, 75% of the tested compounds were active. Among the PubChem hits, synthetic inhibitors were also correctly classified. Accordingly, the model will be applicable to both natural products and synthetic compounds.

Several other computational approaches to predict CYP2D6 inhibition have been published recently^5–7, 34^. However, these *in silico* platforms have not been used to prospectively identify and biologically test new inhibitors. One of these platforms, CypRules^6^, is publicly available.

When tested with our newly found inhibitors and the compounds that were inactive in the CYP2D6 *in vitro* assay, 62% of the active and 83% of the inactive compounds were predicted correctly (for details refer to the Supplementary data Table S10).

As recent studies confirm^11, 24, 35^, bioluminescence-based high-throughput assays are vulnerable to compounds that exhibit a multitude of biological activity on different targets^24^. In addition, it was found that luciferase inhibition can influence the output of the assay^11^. To address this topic and to strengthen the reliability of the new hits, we incorporated an independent control in the workflow to consider a possible inhibition of the detection reaction.

As depicted in Fig. 1, the CYP2D6 inhibition assay consisted of three steps. The CYP reaction *per se* accounted only for the first step, whereas the second one was a de-esterification step and the third step was the luciferase reaction that generated the detection signal^8^. A detection reaction control served to detect interferences of the compounds with the assay setup rather than with the investigated enzyme. This control step could serve as a stop or go signal for further, more laborious investigations, such as e.g. dose-dependent inhibition studies.

For a more in-depth characterization of compounds, time-dependent inhibition studies are recommended in the guidelines on DDI studies of both the European Medicines Agency (EMA) and the Food and Drug Administration (FDA)^36, 37^. The FDA industry guidance for drug interaction studies defines time-dependent inhibition as an inhibitory effect of the compound of interest, which increases over time and is not promptly reversible^37^. This effect cannot be seen when the compound interferes with the reactions involved in signal detection and may lead to a high rate of false positive results.

A possible alternative approach to evaluate the inhibition of the CYP-reaction is to rely on validated HPLC-MS/MS methods^25^. Although their high sensitivity and selectivity^25^ are advantageous, these options require expensive technical equipment and are usually only suitable for low-throughput screenings.

**Figure 9:**
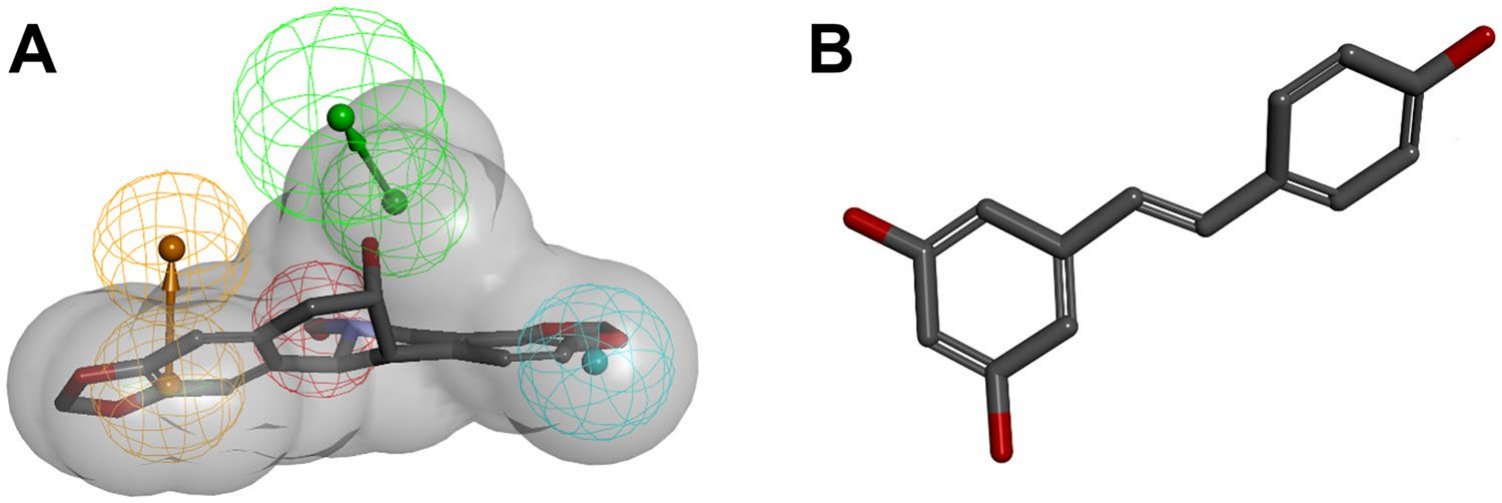
Comparison of the pharmacophore model for CYP2D6 inhibitors with a structurally diverse compound. (**A**) The newly identified compound **21** was found by the model and thus shares the necessary features to act as a strong inhibitor in the CYP2D6 assay. (**B**) **24** is structurally diverse and does not fit into the model, but exhibits an intermediate inhibitory potential on CYP2D6 mainly by interacting with the detection reaction. While it also has aromatic, hydrophobic and hydrogen bonding features, the lack of a positively ionisable nitrogen is obviously contributing to the actual inactivity of **24**.

Pharmacophore models filter compounds that share similar molecular features; therefore, the chemical uniformity of the *in vitro* dataset is increased. Thus, the chance that the so preselected compounds then bind in similar poses or related regions of the targeted enzyme area will be higher. Allosteric binding at the outside of the active site cavity can be widely ignored. Though this selectivity is a benefit of the *in silico* approach, it narrows the compound spectrum and possible inhibitors with differing structures will not be selected for the following *in vitro* testing, due to the lack of physicochemical similarity with known inhibitors (Fig. 9). Furthermore, only single substances and no complex mixtures can be tested *in silico*, as usually most parts of a mixture’s constituents are not known. In addition, synergistic effects are not predictable with this *in silico* approach.

On the contrary to the *in silico* model, the *in vitro* test system used in this study can also be applied to complex mixtures with unknown constituents such as plant extracts, which are frequently investigated in natural product research. In this context, the relevance of a quenching control is even more important (Fig. 7).

In conclusion, the presented combined virtual - *in vitro* workflow may help to screen natural compound libraries fast and reliably for new CYP2D6 inhibitors. Moreover, detailed information on CYP2D6 inhibition is essential when aiming to combine herbal preparations and synthetic drugs at the same time safely.

## Materials and Methods

### Preparation of the *in silico* pre-selection workflow

#### Selection of the dataset

As a test-dataset for the optimization of the pre-selection workflow, data from the PubChem Assay AID891^15^ was used that incorporates 9385 NIH compounds tested for CYP2D6 inhibition. Among the tested compounds, 1623 were active, 6338 were inactive and 1424 showed inconclusive action on CYP2D6. Duplicate entries were removed. Two separate structure data-files (sd-files) were used that consisted *1)* of the active and *2)* of the inactive compounds from the PubChem homepage.

#### Building and screening of 3D-databases

The 3D-databases were built on the basis of the two sd-files (*i.e*. actives and inactives) by using the ‘Build 3D-database’ protocol of the Discovery Studio software, Version 4.0^38^. For each compound, *i.e*. 1623 actives and 6338 inactives in the sd-files, a maximum of 255 conformers was calculated. The resulting 3D-databases were screened with a pharmacophore model^12^ in the ‘Search 3D-database’ protocol of Discovery Studio and a rigid (FAST mode) and a flexible (BEST mode) search was applied on each single 3D-database, respectively.

#### Selection of a workflow based on theoretical quality metrics

In order to quantify the outcome of the pre-selection workflow, we calculated an enrichment factor (EF)^17^ by using the formula EF = (TP/n)/(n_act_/N). TP equals the number of true positive hits that were correctly found by the model, n is the number of compounds in a hit list that fit the model, n_act_ represents the number of active compounds in the whole database and N equals the number of all entries in the validation database. The accuracy (ACC) was calculated by the formula ACC = (TP + TN)/(TP + FP + TN + FN). In this equation, TP equals the number of true positive hits that were found correctly by the model. TN equals the number of true negatives that is the number of inactives not found by the model. False positives (FP) equals the number of hits found in the inactives database and false negatives (FN) equals the number of actives not found by the model^39^.

### Application of the validated pre-selection workflow on a random database

#### Preparation of the natural products 2D-databases

The compounds from the 16 in-house databases were provided by five different university collaborators (Pharmacognosy Department, University of Innsbruck, Austria; Department of Pharmaceutical Sciences, Federal University of Santa Catarina, Florianopolis, Brazil; Pharmacognosy Department, University of Graz, Austria; Department of Pharmacognosy, University of Vienna, Austria; Griffith Institute for Drug Discovery, Griffith University, Australia). One database was published at the SPECS-homepage (accessed in January 2016).

#### Building and screening of 3D-databases

The 3D-databases were generated by applying the software settings from the validation of the pre-selection workflow, namely building the database in the BEST mode and creating a maximum amount of 255 conformers for each entry in the database. For screening of the built up 3D-database, we employed the rigid search function in Discovery Studio.

### Selection of the *in silico* hits for *in vitro* testing

In order to obtain information on already existing CYP2D6 inhibition data of the compounds in the virtual hit list, we examined literature by searching the SciFinder^19^ database for key-words like: cytochrome P450, 2D6 and CYP2D6 and combinations thereof. Compounds of natural origin (chemically unmodified) lacking CYP2D6 inhibition data were selected for the screening.

### Evaluation of the CYP2D6 inhibition *in vitro*

*Chemicals and reagents*. Quinidine (**25**) was purchased from Sigma-Aldrich Chemicals (St. Louis, MO, USA) as European Pharmacopoeia (EP) Reference Standard. The compounds that were selected for *in vitro* testing were kindly provided from the Department of Pharmacognosy, University of Innsbruck, Austria, *i.e*. **1**–**3**, and the Griffith Institute for Drug Discovery, Griffith University, Australia, *i.e*. **4**–**7** and **21**–**23**. Compounds **8**–**20** and **24** were purchased from SPECS (Zoetermeer, NL). The purity of the compounds that were purchased from SPECS was > 95% for 8 compounds *i.e*. **9**, **10**, **12**, **13**, **17**, **19, 20** and **24**, 95% for compound **8** and 90% for 5 compounds *i.e*. **11**, **14**, **15**, **16** and **18**. More information on the compounds from the university sources can be found in the Supplementary data section. ME-luciferin-EGE, luciferin-EGE, NADPH-regeneration system and the luciferin detection reagent with esterase were purchased from Promega Corporation (Madison, WI, USA). Recombinant human CYP2D6 BACULOSOMES Plus Reagent came from Life Technologies (Carlsbad, CA, USA). All other reagents were of the highest purity commercially available or at least HPLC grade.

#### Equipment

All *in vitro* experiments were performed in sterile, white opaque, Nunclon Delta Surface 96-well plates. Luminogenic signals were detected with a Tecan infinite F200 PRO plate reader (integration time of 1 s per well).

#### Compound preparation

All screening compounds used for *in vitro* testing were dissolved in DMSO after arrival. Aliquots of 8 µl of a 10 mM DMSO stock solution were stored at −20 °C until use at the experiment day. For each experiment, one aliquot was thawed and diluted with 192 µl of luciferase-free water to give a 400 µM working dilution that was stored on ice until use. **25** was dissolved in absolute ethanol to render a 20 mM stock solution that was prepared in luciferase-free water at the experiment day to a working dilution of 4 µM.

#### CYP2D6 inhibition pre-screen assay at a concentration of 100 µM

Incubation of CYP2D6 for the pre-screen inhibition assay (Promega) was performed by filling each well of a white 96-well plate with 12.5 µl of either the 400 µM test compound solution (samples), 4 µM working dilution of compound **25** (positive control), 4% DMSO (solvent control) or luciferase free water (minus P450 control), all in triplicates. A 4× reaction mixture was prepared, composed of 15 µl of the 120 µM ME-luciferin-EGE stock solution, 500 µl of the 1 M KPO_4_ buffer at pH 7.4 and 710 µl luciferase free water (final volume of 1225 µl). In the three minus P450 control wells, 12.25 µl of the 4× reaction mixture was added. Then, 24.25 µL (24.25 pmol) of recombinant human CYP2D6 baculosomes (Life Technologies) was added to the remaining 4× reaction mixture; 12.5 µl of the solution was transferred into the other wells. The plate was incubated for 10 minutes. The CYP2D6 enzyme reaction was initiated by the addition of 25 µl of 2× NADPH regeneration system (2.6 mM NADP^+^, 6.6 mM glucose-6-phosphate, 6.6 mM MgCl_2_ and 0.8 U/ml glucose-6-phosphate dehydrogenase buffered in 0.1 mM sodium citrate at pH 5.5; Promega). After 45 minutes incubation, the enzyme reaction was stopped by the addition of 50 µl of the luciferin detection reagent, which contained also the esterase for the generation of the luminescence signal. The plate was incubated in the dark for 20 minutes in order to stabilize the signal measured by a Tecan infinite F200 PRO plate reader. The final assay concentrations are listed in the Supplementary data section (Supplementary Table S4). The maximum allowed DMSO concentration in the final reaction mix was 1% according to the manufacturer’s protocol. The experiment was performed at room temperature and under prevention of excessive UV-light at minimum on two independent days (n = 2).

#### P450-Glo detection control

For the P450-Glo detection control, wells were filled with either 12.5 µl of the 400 µM working dilution of the test compounds (samples), the 4 µM working dilution of compound **25** (positive control), 4% DMSO (solvent control) or luciferase free water (background luminescence), respectively in triplicates. We then added 12.5 µl 4× reaction mixture (5 µl 1.5 mM luciferin-EGE stock solution, 500 µl 1 M KPO_4_ buffer and 745 µl luciferase free water) to the wells. Finally, 25 µl of the 2× NADPH regeneration system was added. In order to initiate the detection reaction, 50 µl of the reconstituted luciferin detection reagent containing the esterase was added to the samples, positive and solvent control wells. This step was followed by 20 minutes incubation in the dark for signal stabilization. In the wells reserved for background luminescence, luciferase-free water was added. Measurements were performed on a Tecan infinite F200 PRO plate reader on two independent days (n = 2) at minimum.

#### IC_50_ determination of active and weakly active compounds

Concentration-dependent CYP2D6 inhibition assays were performed by preparing a 5-step dilution series of the 400 µM compound solutions. This was performed for the strong 1:10 and for the weak inhibitors 1:3 from the highest to the lowest concentrations of 4 nM and 0.16 µM, respectively. The concentrations in the final reaction volume were 100–0.001 µM for the active and 100–0.4 µM for the weakly active compounds, respectively. After this dilution step, 12.5 µl of each dilution was transferred to the assay plate. The assay was performed as described above. The DMSO concentration was kept constant for all concentrations with a maximum of 1%. The experiments were performed on three independent experimental days (n = 3).

#### Quantification of the luminogenic signal

Raw data obtained from Tecan infinite F200 PRO plate reader was processed by calculating total luminescence (% of untreated control) by applying the formula: raw data − (background luminescence) * 100/mean of negative control.

#### Calculation of the IC_50_ values

IC_50_ values were calculated by nonlinear regression analysis using the equation Y = Bottom + (Top-Bottom)/(1 + 10^((LogIC50-X)*HillSlope))^ from Graph Pad Prism Version 6.05 for Windows, (GraphPad Software, La Jolla California USA, www.graphpad.com). All available 6 data-points of the concentration-response curve were included, only compound **18** included 4 of 6 available points due to non-monotonic response at low-level concentrations^29^.

#### Statistical analysis of the *in vitro* dataset

For the analysis of the P450-Glo CYP2D6 inhibition pre-screen assay, compounds **21** and **24** were compared to the DMSO control (Fig. 7A). For the P450-Glo detection control, the results for compound **1**–**24** were compared to the DMSO control group (Fig. 7B, Table 1). A one-way ANOVA followed by Dunnett’s multiple comparisons test was performed. For the statistical analysis, GraphPad Prism version 6.05 for Windows (GraphPad Software, La Jolla California USA, www.graphpad.com) was used. A p-value of < 0.05 was used to indicate significance. The *in vitro* dataset of the P450-Glo CYP2D6 inhibition pre-screen assay (Fig. 7A) and of the P450-Glo detection control (Fig. 7B and Table 1) served as a basis for the statistical analysis.

### Preparation of a docking workflow for CYP2D6

In order to establish a docking workflow for the inhibitors, we used the wizard of the docking software GOLD 5.2, chose GoldScore as the fitness function and loaded the crystal structure of CYP2D6 co-crystallized with the inhibitor ajmalicine (4WNT)^40, 41^. As we set up the protein for docking, we added 7571 hydrogens, deleted 126 waters and deleted the co-crystallized molecule ajmalicine from the active site cavity. We defined an area of 10 Å around the coordinates 7.14, 31.46 and −73.09 in the x, y and z-axis, respectively, as the binding site for the docking process. Early termination was not allowed and the internal energy offset was used. The genetic algorithm search options were kept default and the search efficiency was set at 100%. To validate the docking and identify the root mean square deviation from the core molecule, we selected the extracted core molecule ajmalicine and an independently generated ajmalicine molecule. More information on the re-docking process can be found in the Supplementary data.

### Visualization of the ligand binding using the docking model

For the prediction of the protein-ligand interactions in the active site cavity, we applied the docking settings that proved to be most suitable from the re-docking process (see Supplementary data) on the 3 strongest inhibitors (**21**, **19** and **9**) by using the genetic algorithm implemented in the software GOLD^40, 41^. The corresponding docking solution of each inhibitor with the highest gold score was selected for the visualization and analysis of the 3D protein-ligand interactions, which was achieved by using LigandScout version 3.12^42^.

## Electronic supplementary material

Supplementary Info File #1
