# Supplementary material for: A combinatorial approach for the discovery of cytochrome P450 2D6 inhibitors from nature
Source: Sci Rep. 2017 Aug 14;7:8071. doi: 10.1038/s41598-017-08404-0 (PMC5556109; doi:10.1038/s41598-017-08404-0)
Supplement: Supplementary file 1 — Supplementary Info File #1 [file 41598_2017_8404_MOESM1_ESM.pdf]

# Supplementary Data

# A combinatorial approach for the discovery of cytochrome P450 2D6 inhibitors from nature

Johannes Hochleitner[1], Muhammad Akram[2], Martina Ueberall[1], Rohan A. Davis[3], Birgit Waltenberger[4], Hermann Stuppner[4], Sonja Sturm[4], Florian Ueberall[1+], Johanna M. Gostner[1*], Daniela Schuster[2+*]

1 Division of Medical Biochemistry, Center for Chemistry and Biomedicine, Medical University of Innsbruck, Austria
2 Institute of Pharmacy / Pharmaceutical Chemistry, Computer Aided Molecular Design Group and Center for Molecular Biosciences Innsbruck (CMBI), University of Innsbruck, Austria
3 Griffith Institute for Drug Discovery, Griffith University, Brisbane, QLD 4111, Australia
4 Institute of Pharmacy / Pharmacognosy and Center for Molecular Biosciences Innsbruck (CMBI), University of Innsbruck, Austria
+ These authors contributed equally to this work
* Corresponding Author

## *Table of contents*

*In silico* dataset for the screening of novel CYP2D6 inhibitors. .... 2
CYP2D6 inhibition of virtual hits already reported in the literature .... 3
Information on the compounds tested *in vitro* .... 4
Incubation conditions for the *in vitro* assay and the luminescence quenching control .... 5
Concentration-response curves of the strongest inhibitors .... 7
Establishment of the re-docking process .... 8
Interactions of the three most potent inhibitors in the active site cavity of 4WNT .... 9
Comparison of the docking poses in different PDB structures .... 10
CypRules performance comparison .... 16
References .... 17

# *In silico* dataset for the screening of novel CYP2D6 inhibitors

## Building of the 3D-database

Every single 2D-database was treated and built up separately using the BEST mode and creating a maximum of 255 conformers for each single substance in the 2D-database, according to the findings from the validation of the *in silico* workflow. From all of the 2,147 single substances that make up the 17 2D-databases we could recover 86 % *i.e.* 1,847 compounds that account for the 3D-databases, whereas 11 databases were built without declines and 6 databases were built with an overall loss of 300 compounds (Supplementary Table S1).

## Screening of the 3D-database

We screened the 17 3D-databases with overall 1,847 compounds and applied a rigid search using the pharmacophore model for CYP2D6 inhibitors[1] that proved to be an appropriate tool in the preceding validation workflow. From the screened 1,847 compounds in 17 3D-databases, we identified 4.1 % *i.e.* 75 compounds that fitted the model for potential CYP2D6 inhibitors and that were located in 6 different 3D-databases. In 10 databases we were not able to identify compounds that fitted the pharmacophore model for CYP2D6 inhibitors (Supplementary Table S1).

**Supplementary Table S1. *In silico* dataset for the screening of novel CYP2D6 inhibitors.**

| University Collaborators | Databases | Input 2D-database | Output 3D-database | Hits Total | Hits selected |
|---|---|---|---|---|---|
| Pharmacognosy_IBK | Birgit_C13_AR(+)_Birgit_MV26 | 2 | 2 | - | - |
| Florianopolis_Brazil | Florianopolis_Falkenberg | 3 | 3 | - | - |
| Pharmacognosy_Graz | Polyacetylenes_from_Notopterygium | 5 | 5 | - | - |
| Pharmacognosy_IBK | Naturstoffe SKC-Files | 7 | 7 | - | - |
| Pharmacognosy_Vienna | lilo_krenn_compounds | 13 | 13 | - | - |
| Rohan_Davis | Rohan_Davis_Structures_II_checked | 14 | 13 | 5 | 3 |
| Pharmacognosy_IBK | Semisynthetika SKC-Files_CA_Isis | 20 | 20 | - | - |
| Pharmacognosy_Graz | Bupleurum_compounds | 21 | 21 | - | - |
| Pharmacognosy_Graz | Polyacetylenes_from_Oplopanax | 29 | 29 | - | - |
| Pharmacognosy_IBK | Semisynthetika CDX-Files | 48 | 48 | 3 | - |
| Pharmacognosy_IBK | Semisynthetika SKC-Files_AK_Isis | 51 | 51 | - | - |
| Pharmacognosy_IBK | Cdx-Files_Naturstoffe+Synthetika | 56 | 56 | 3 | - |
| Pharmacognosy_IBK | Naturstoffe CDX-Files | 75 | 72 | 6 | 3 |
| Pharmacognosy_Vienna | Atanasov_Vienna_NAT_library | 114 | 109 | - | - |
| Pharmacognosy_IBK | Substanzen_Pharmakognosie | 236 | 221 | 9 | - |
| Rohan_Davis | Rohan_Davis_NP_library_140704_cleaned | 576 | 429 | 10 | 3 |
| SPECS | Specs_NP_1mg_Jan2016_BEST_255 | 877 | 748 | 39 | 14 |
| | **Sum** | **2,147** | **1,847** | **75** | **23** |
| | **Recovery (%)** | **100.0** | **86.0** | **4.1** | **1.2** |

## CYP2D6 inhibition of virtual hits already reported in the literature

In the course of the screening of the various databases we found 75 hits that fitted the pharmacophore model. From these hits, 23 were selected for the *in vitro* screening. We selected the hits according to non-available CYP2D6 inhibition data, plant origin and availability. The 52 not-tested hits included some duplicate entries and were composed of natural, synthetic or semisynthetic compounds. Focusing on the compounds from plant origin, highly potent and well-known CYP2D6 inhibitors were found by the pharmacophore model, like ajmalicine and quinidine. On the one hand, this was expected because some of these compounds have been used for model generation[1] already. On the other hand, additional active compounds were found, which underlines the power of the *in silico* pre-selection step (Supplementary Table S2).

**Supplementary Table S2. Inhibitory potency of already tested inhibitors found by the model.**

| CAS | Name | Database | Inhibitory potency | Unit | $IC_{50}$/Ki | Classification | Reference |
|---|---|---|---|---|---|---|---|
| 483-04-5 | Ajmalicine | 1 | 2.3 | nM | $IC_{50}$ | active | [2] |
| 56-54-2 | Quinidine | 1 | 7.5 | nM | $IC_{50}$ | active | in-house |
| 130-86-9 | Protopine | 1 | 78 | nM | Ki | active | [3] |
| 65-19-0 | Yohimbine | 1 | 0.062-0.72 (substrate dependent) | µM | $IC_{50}$ | active | [4] |
| 3520-14-7 | Tetrahydro palmatine | 1 | 3.04 ± 0.26 | µM | $IC_{50}$ | active | [5] |
| 6018-39-9 | Corypalmine | 1 | 1-10 | µM | $IC_{50}$ | active | [6] |
| 485-71-2 | Cinchonidine | 1 | 10 | µM | $IC_{50}$ | active | [7] |
| 130-95-0 | Quinine | 1 | 10 | µM | $IC_{50}$ | active | [7] |
| 2182-14-1 | Vindoline | 2 | 15.9 | µM | $IC_{50}$ | active | [2] |
| 518-69-4 | Corydaline | 1 | 64.5 ± 9.8 (w/ preincubation) | µM | $IC_{50}$ | weak | [8] |
| 518-69-4 | Corydaline | 1 | 115.9 ± 7.6 (w/o preincubation) | µM | $IC_{50}$ | inactive | [8] |
| 10605-02-4 | Palmatine chloride | 1 | 92.6 | µM | - | weak | [9,10] |
| 115-53-7 | Sinomenine | 2 | no inhibition at 50 | µM | - | inactive | [11] |
| 128-62-1 | Noscapine | 1 | >100 | µM | - | inactive | [12] |
| 2688-77-9 | Laudanosine | 2 | stated as inhibitor | - | - | unclear | [13] |
| 482-74-6 | Cryptopine | 2 | stated as inhibitor | - | - | unclear | [6] |

databases: **1** Pharmacognosy Innsbruck, **2** SPECS

## Information on the compounds tested *in vitro*

**Supplementary Table S3. All compounds tested *in vitro.* The numbers assigned are the same as in the manuscript.**

| Index | SciFinder CAS | Molecular weight incl. salts [g] | Name | Salt data |
|---|---|---|---|---|
| **1** | 5629-60-7 | 368.43 | Pteropodine | |
| **2** | 5171-37-9 | 368.43 | Isopteropodine | |
| **3** | 29550-24-1 | 353.41 | Thalictrivacine | |
| **4** | 89647-69-8 | 531.48 | Pistillarin | TFA |
| **5** | 1620102-34-2 | 418.32 | Secofascaplysic acid | TFA |
| **6** | 61-25-6 | 375.85 | Papaverine | HCl |
| **7** | 2468-21-5 | 336.43 | (+)-Catharanthine | |
| **8** | 4837-77-8 | 526.42 | Venenatine methiodide | CH3I |
| **9** | 4781-58-2 | 487.56 | (S)-Norreticuline | tosic acid |
| **10** | 5001-20-7 | 336.45 | (-)-Venalstonine | |
| **11** | 3122-95-0 | 379.88 | (+)-Laudanidine | HCl |
| **12** | 477-30-5 | 371.42 | Colcemid | |
| **13** | 6871-44-9 | 420.94 | Echitamine | HCl |
| **14** | 175274-51-8 | 327.37 | Spinosine | |
| **15** | 73870-36-7 | 377.92 | Des-N,O-dimethylarmepavine | HCl |
| **16** | 559-48-8 | 380.44 | (-)-Kopsine | |
| **17** | 4939-81-5 | 322.42 | (+)-Condylocarpine | |
| **18** | 25651-04-1 | 327.37 | Bracteoline | |
| **19** | 485-19-8 | 429.81 | (+)-Reticuline | HClO4 |
| **20** | 1936-18-1 | 327.37 | (+)-Salutaridine | |
| **21** | 476-32-4 | 353.37 | (+)-Chelidonine | |
| **22** | 4429-63-4 | 372.89 | Tabersonine | HCl |
| **23** | 4963-01-03 | 368.43 | Isomitraphylline | |
| **24** | 501-36-0 | 228.23 | Resveratrol | |
| **25** | 6591-63-5 | 782.94 | 2 Quinidine | $H_2SO_4$, 2 $H_2O$ |

### Preparation of the compounds from the university collaborators-UIBK

The compounds **1** and **2** were isolated at the Institute of Pharmacy / Pharmacognosy, University of Innsbruck. The identification of the structure was performed by mass and NMR spectroscopy as well as by comparison (TLC, HPLC) with authentic samples. The compound **3** was isolated and identified as described previously[14].

### Preparation of the compounds from the university collaborators-Rohan A. Davis

The isolation and identification of compounds **4** and **5** has been described elsewhere[15,16]. The compounds **6**, **7**, **21**, **22** and **23** were purchased from PhytoLab (www.phytolab.com).

# Incubation conditions for the *in vitro* assay and the luminescence quenching control

**Supplementary Table S4. Reaction volumes used of the CYP2D6 inhibition assay and the detection control of the P450-Glo reaction.**

| **P450-Glo CYP2D6 inhibition assay (Promega)** | | [µl] |
|---|---|---|
| 1 | test compound | 12.5 |
| 2 | enzyme/substrate mix (CYP2D6/ME-luciferin-EGE) | 12.5 |
| 3 | NADPH regeneration system | 25 |
| 4 | detection reagent | 50 |
| | total volume | 100 |

| **inhibition control of the P450-Glo detection reaction** | | [µl] |
|---|---|---|
| 1 | test compound | 12.5 |
| 2 | enzyme/substrate mix (no CYP2D6/ luciferin-EGE) | 12.5 |
| 3 | NADPH regeneration system | 25 |
| 4 | detection reagent | 50 |
| | total volume | 100 |

**Supplementary Table S5. Incubation conditions for the P450-Glo CYP2D6 inhibition pre-screen and for the P450-Glo detection reaction control**

| | **P450-Glo CYP2D6 inhibition pre-screen** | | **P450-Glo detection reaction** |
|---|---|---|---|
| components | final concentrations for CYP2D6 reaction | addition of detection reagent | |
| test compound | 100 µM | 50 µM | 50 µM |
| ME-luciferin-EGE | 30 µM | ~15 µM | - - |
| luciferin-EGE | - - | ~0.75 µM | 0.75 µM |
| $KPO_4$ buffer at pH 7.4 ($K_2HPO_4$/$KH_2PO_4$, 0.8/0.2) | 100 mM | 100 mM | 100 mM |
| rhCYP2D6 baculosomes | 0.25 pmol | 0.125 pmol | - - |
| NADP+ | 1.30 µM | 0.65 µM | 0.65 µM |
| glucose-6-phosphate | 3.30 µM | 1.15 µM | 1.15 µM |
| $MgCl_2$ | 3.30 µM | 1.15 µM | 1.15 µM |
| glucose-6-phosphate dehydrogenase | 0.40 U/ml | 0.20 U/ml | 0.20 U/ml |
| sodium citrate pH 5.5 | 0.05 mM | 0.025 mM | 0.025 mM |
| DMSO concentration | 1 % | 0.5 % | 0.5 % |
| detection reagent | - | 50 µl | 50 µl |
| total volume incl. detection reagent | - | 100 µl | 100 µl |
| reaction volume | 50 µl | - | 100 µl |

**Incubation times**

| | **P45-Glo CYP2D6 inhibition pre-screen** | **P450-Glo detection reaction** |
|---|---|---|
| pre-incubation | 10 min | - - |
| CYP2D6 enzyme reaction | 45 min | - - |
| stabilization of luminogenic signal | 20 min | 20 min |

**Plate reader settings**

| | |
|---|---|
| signal detection integration time | 1s |
| attenuation | automatic |

## Concentration-response curves of the strongest inhibitors

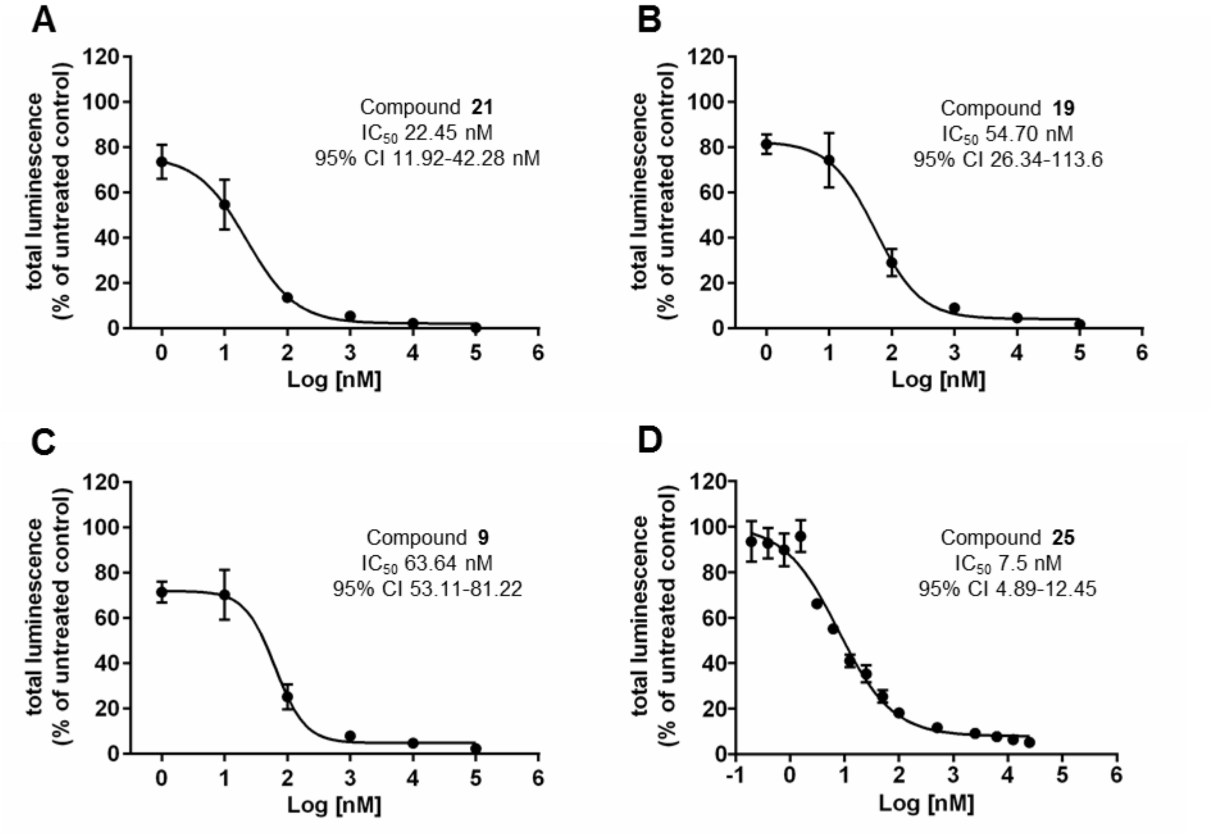


**Supplementary Figure S1. Concentration-response curves for the three most potent inhibitors.** $IC_{50}$ values were determined for the compounds, which showed a total luminescence greater than 50 % at 100 µM in the inhibition assay for P450-Glo CYP2D6. The compounds **21** (A), **19** (B) and **9** (C) turned out as strong inhibitors of CYP2D6 activity as their $IC_{50}$ values were in the lower nanomolar range. The $IC_{50}$ value for the model inhibitor **25** was comparable to the values reported in the literature (D). The error bars indicate the standard error of mean of triplicate measurements obtained in three independent experiments.

## Establishment of the re-docking process

In order to predict the binding pose of an inhibitor in the active site cavity, the program settings were validated by re-docking of ligands to a CYP2D6 structure that was solved as a co-crystallized complex with inhibitors in the active site cavity by Wang et al (PDB entry 4WNT[19]). The re-docking helps to identify the software settings with which the crystallographic and thus known binding pose can be reproduced by a computational docking method within a range of a root mean square deviation (RMSD) at or below 2 Å. The re-docking experiments were performed using the genetic algorithm implemented in GOLD[17,18]. Independent re-docking models for each of the crystal structures 4WNT[19], 4WNU[19] and 4XRZ[20] were generated that exhibit a RMSD of 0.19, 0.95 and 0.60 Å for the core to the co-crystallized ligand compared to the re-docked ligand, respectively. Furthermore, each re-docking model was challenged with a self-generated ligand to avoid bias by using the bioactive conformation of the ligand for re-docking. For all three crystal structures *i.e.* 4WNT, 4WNU and 4XRZ, the RMSD of the core, co-crystallized ligand to the self-generated re-docked ligand was 0.37, 0.51 and 0.74, respectively. The docking and re-docking workflow obtained from 4WNT, with ajmalicine co-crystallized in the active site cavity (Supplementary Fig. S2) turned out to be best suited for the further studies.

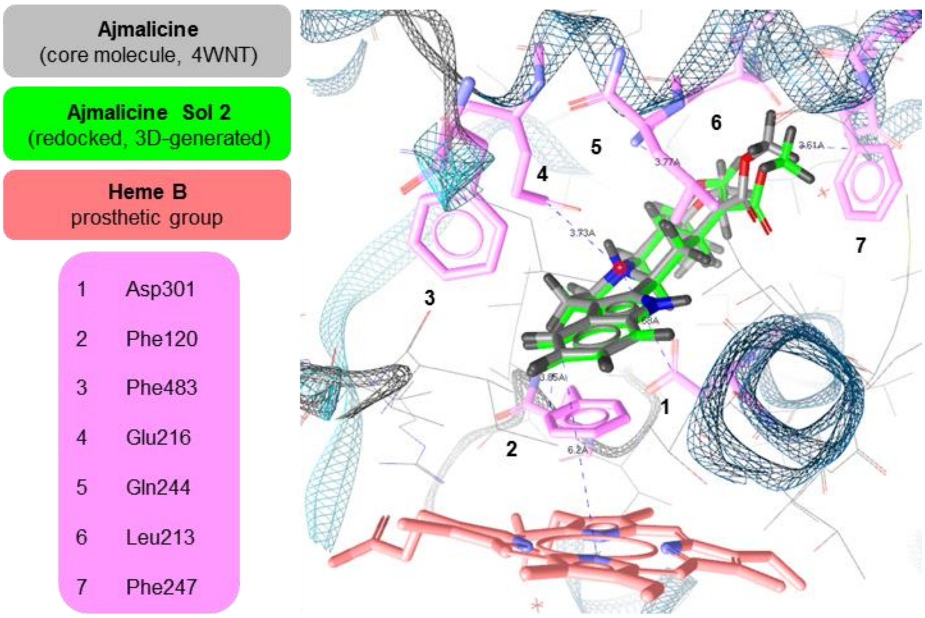


**Supplementary Figure S2. Visualization of ajmalicine in the active site cavity of CYP2D6 with a docking model.** The docking model for CYP2D6 was based on the crystal structure co-crystallized with the inhibitor ajmalicine (PDB code 4WNT), having a RMSD value below 0.4 Å of the core molecule to the re-docked molecule. The amino acids highlighted in magenta listed on the left side were found to be important interaction and binding partners in previous studies. The prosthetic heme-b group is highlighted in red.

# Interactions of the three most potent inhibitors in the active site cavity of 4WNT

**Supplementary Table S6. The three most potent inhibitors that were docked in the active site cavity of 4WNT showed the following interactions with the macromolecule:**

| **Chelidonine** | | | |
|---|---|---|---|
| ligand | enzyme-AA | mode of interaction | distance Å |
| Benzene ring | Phe120 | Van der Waals | 3.1 |
| Protonated N | Glu216 | ionic | 3.6 |
| | Gln244 | electrostatic | 3.8 |
| | Ser304 | H-bond | 4.4 |
| Phenantridin-6-ol | Glu216 | H-bond | 2.8 |
| Dioxolo | Asp301 | H-bond | 4.5 |
| **Reticuline** | | | |
| ligand | enzyme-AA | mode of interaction | distance Å |
| Benzene ring (isoquinoline) | Phe120 | Van der Waals | 3.6 |
| Protonated N | Glu216 | ionic | 3.1 |
| Hydroxyphenyl-OH | Asp301 | H-bond | 2.3 |
| Isoquinoline-7-OH | Ser304 | H-bond | 4.7 |
| **(S)-Norreticuline** | | | |
| ligand | enzyme-AA | mode of interaction | distance Å |
| Benzene ring | Phe120 | Van der Waals | 3.8 |
| Protonated N | Glu216 | ionic | 5.1 |
| | Asp301 | ionic | 3.3 |
| Isoquinoline-7-OH | Gln244 | H-bond | 3.6 |
| | Ser304 | H-bond | 4.6 |

## Comparison of the docking poses in different PDB structures

**Supplementary Figure S3. Comparison of the docking poses of the three most potent inhibitors in different PDB structures:** The docking model selected in the redocking process was used for the prediction of the potential binding poses of the newly found inhibitors. Figure S3 shows the compounds **21** (I), **19** (II) and **9** (III), which were the three most potent inhibitors in the *in vitro* screening. Each compound was docked in the active site cavity of the PDB structures 4WNT[19], 4WNU[19] and 4XRZ[20], respectively. For a better comparison, the docked inhibitor is presented on the left sides, whereas the core and the redocked core molecule of the corresponding crystal structure can be found on the right sides (I, II and III_A-F). The docked inhibitors are colored in grey/light blue, the heme-moiety in yellow and the amino acids in magenta. The numbers indicate the follwing amino acids: 1-Ser304, 2-Asp301, 3-Phe120, 4-Phe247, 5-Glu216, 6-Leu213, 7-Gln244 and 8-Phe483.

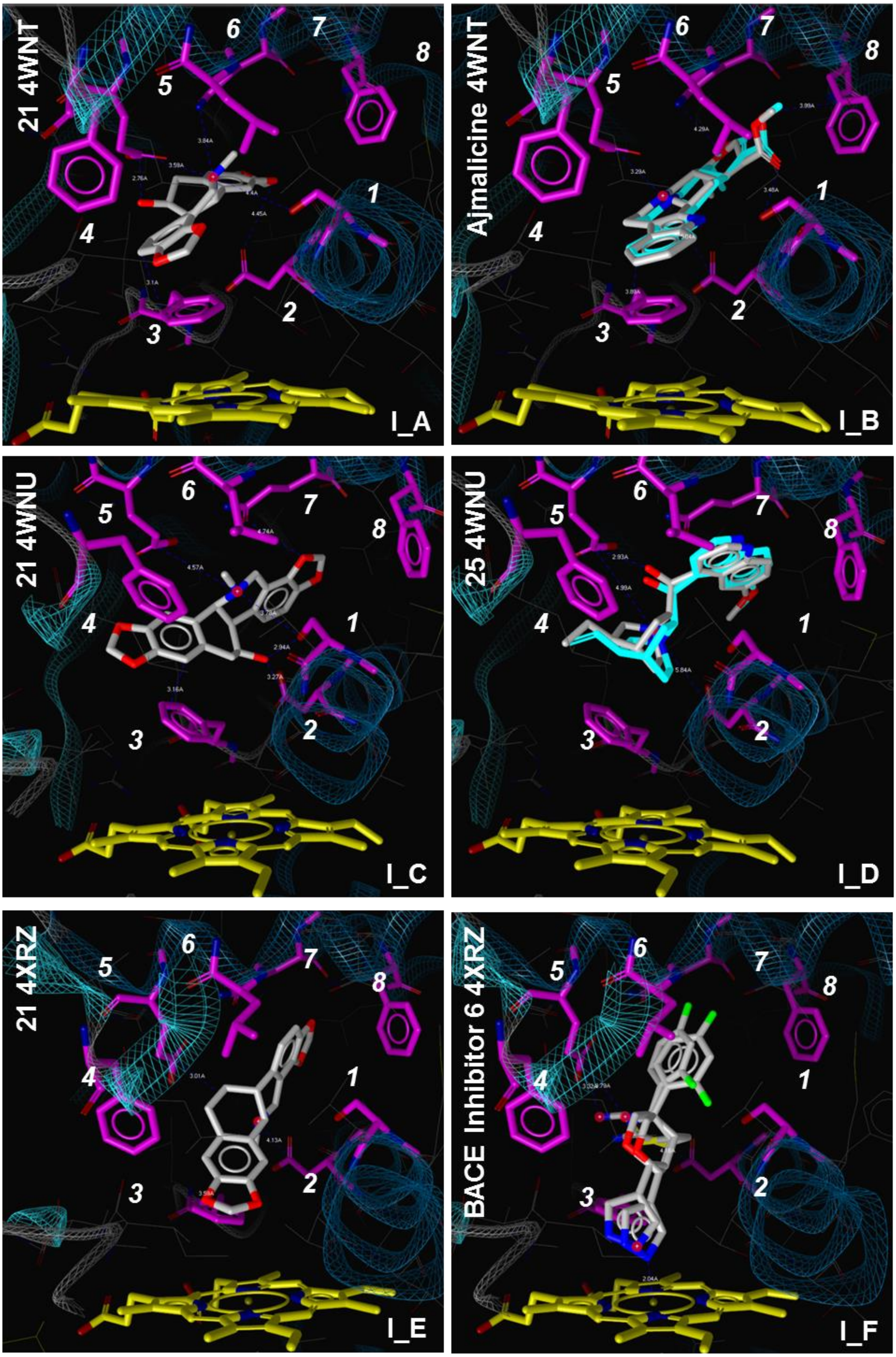
21 4WNT
I_A
Ajmalicine 4WNT
I_B
21 4WNU
I_C
25 4WNU
I_D
21 4XRZ
I_E
BACE Inhibitor 6 4XRZ
I_F
1
2
3
4
5
6
7
8

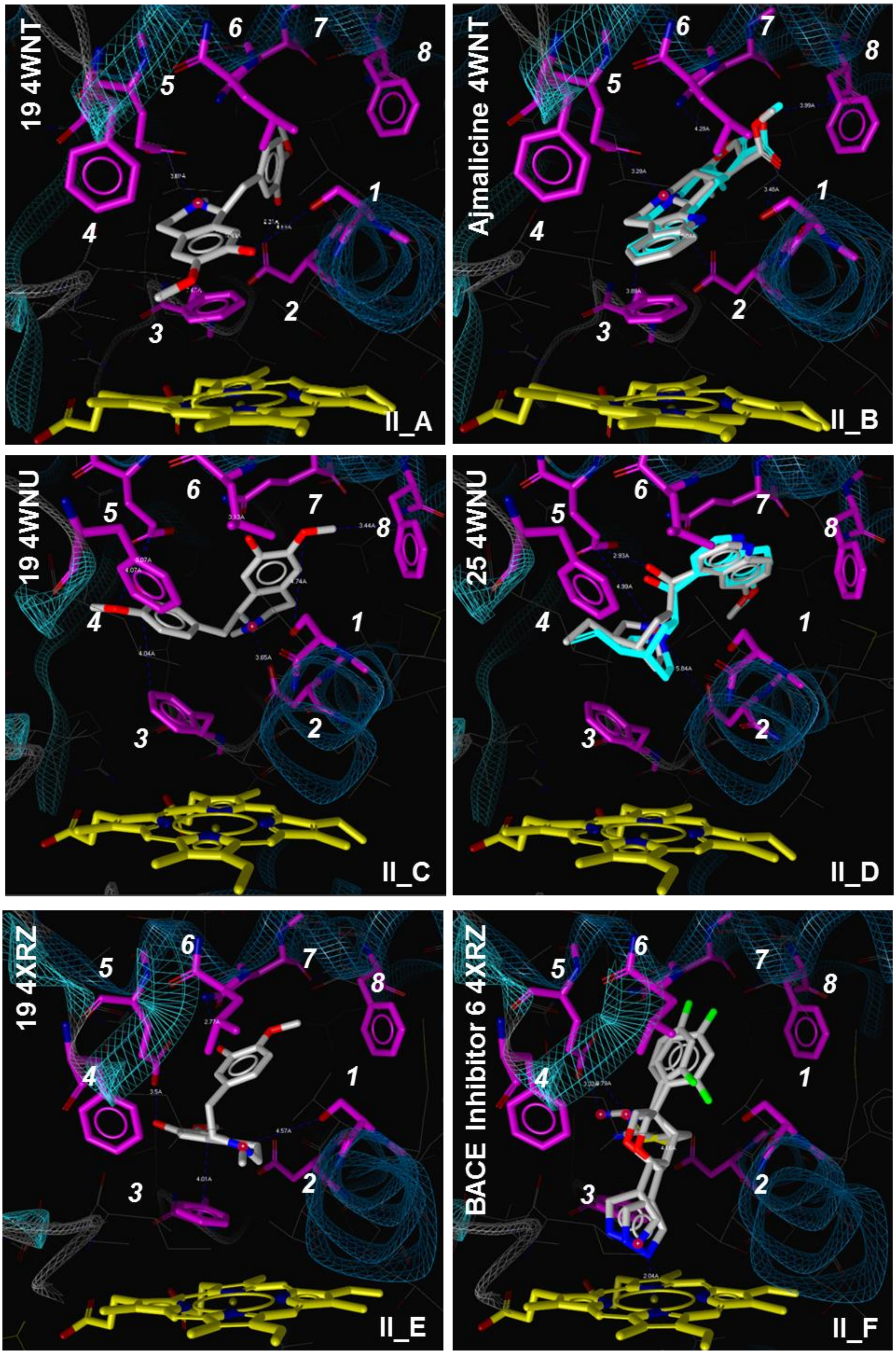
19 4WNT
II_A
Ajmalicine 4WNT
II_B
19 4WNU
II_C
25 4WNU
II_D
19 4XRZ
II_E
BACE Inhibitor 6 4XRZ
II_F

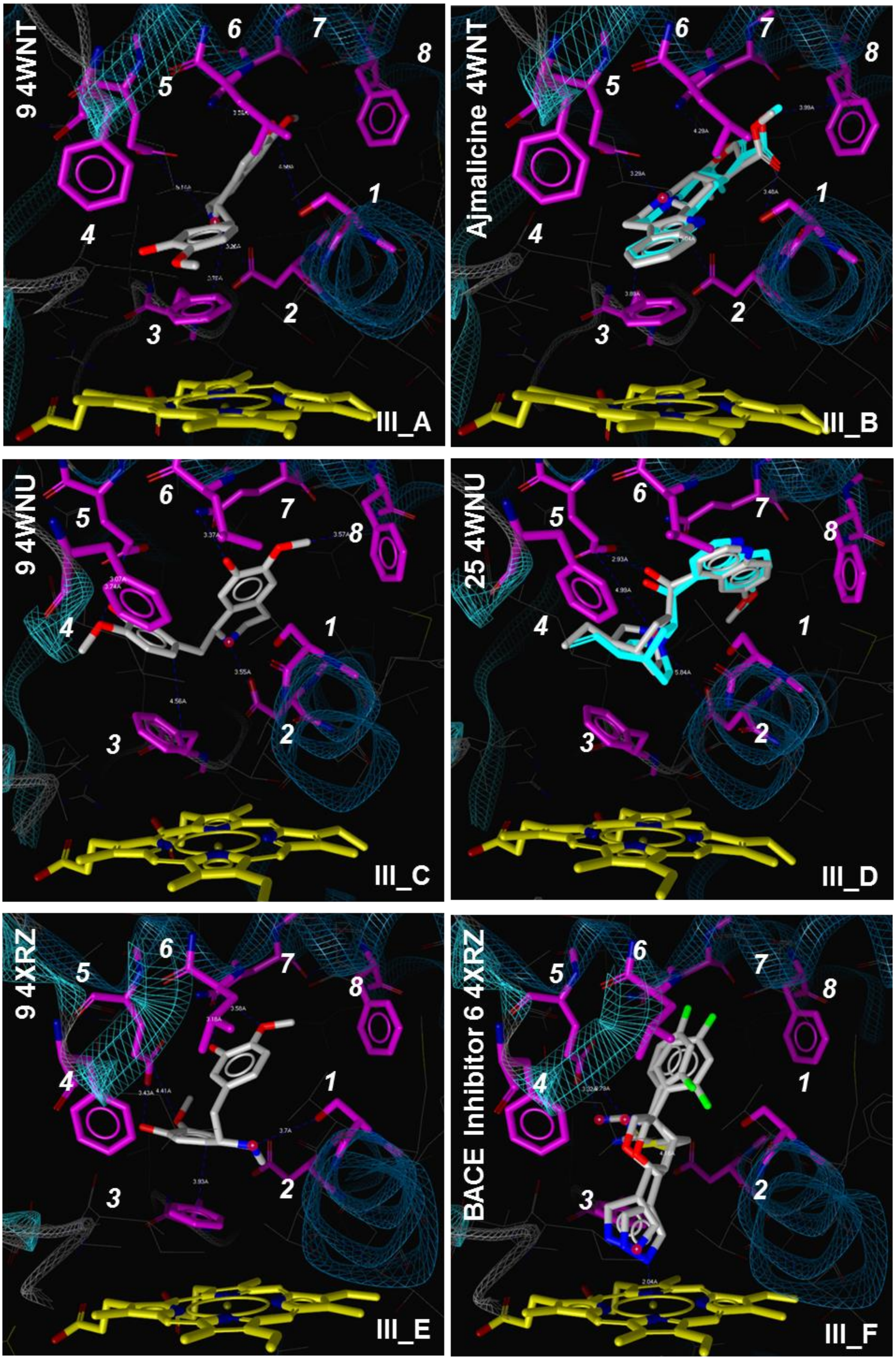
9 4WNT
III_A
Ajmalicine 4WNT
III_B
9 4WNU
III_C
25 4WNU
III_D
9 4XRZ
III_E
BACE Inhibitor 6 4XRZ
III_F
1
2
3
4
5
6
7
8

**Supplementary Table S7. Compound 21 was docked in three different PDB entries. The inhibitor-enzyme interactions are listed and consensus interactions are highlighted in bold.**

| **21 docked in 4WNT** | | | |
|---|---|---|---|
| **ligand** | **enzyme-AA** | **mode of interaction** | **iistance Å** |
| benzene ring | **Phe120** | vdW | 3.1 |
| protonated N | **Glu216** | ionic | 3.6 |
| | Gln244 | electrostatic | 3.8 |
| | Ser304 | H-bond | 4.4 |
| phenantridin-6-ol | Glu216 | H-bond | 2.8 |
| dioxolo | **Asp301** | H-bond | 4.5 |

| **21 docked in 4WNU** | | | |
|---|---|---|---|
| **ligand** | **enzyme-AA** | **mode of interaction** | **distance Å** |
| benzene ring | **Phe120** | vdW | 3.2 |
| protonated N | **Glu216** | ionic | 4.6 |
| | Ser304 | H-bond | 3.8 |
| phenantridin-6-ol | Ser304 | H-bond | 2.9 |
| | **Asp301** | H-bond | 3.3 |
| dioxolo | Gln244 | H-bond | 4.7 |

| **21 docked in 4XRZ** | | | |
|---|---|---|---|
| **ligand** | **enzyme-AA** | **mode of interaction** | **distance Å** |
| benzene ring | **Phe120** | vdW | 3.6 |
| protonated N | **Asp301** | ionic | 4.1 |
| phenantridin-6-ol | **Glu216** | H-bond | 3.0 |

**Supplementary Table S8. Compound 19 was docked in three different PDB structures. The inhibitor-enzyme interactions are listed below and the consensus interactions are highlighted in bold.**

| **4WNT** | | | |
|---|---|---|---|
| **ligand** | **enzyme-AA** | **mode of interaction** | **distance Å** |
| benzene ring (isoquinoline) | **Phe120** | vdW | 3.6 |
| protonated N | **Glu216** | ionic | 3.1 |
| hydroxyphenyl-OH | Asp301 | H-bond | 2.3 |
| isoquinoline-7-OH | **Ser304** | H-bond | 4.7 |

| **4WNU** | | | |
|---|---|---|---|
| **ligand** | **enzyme-AA** | **mode of interaction** | **distance Å** |
| benzene ring | **Phe120** | vdW | 4.0 |
| methoxyphenyl-O | **Glu216** | H-bond | 4.1 |
| hydroxyphenyl-OH | **Glu216** | H-bond | 3.1 |
| protonated N | Asp301 | ionic | 3.7 |
| isoquinoline-7-OH | Gln244 | H-bond | 3.1 |
| isoquinoline-6-methoxy (O) | **Ser304** | H-bond | 4.7 |
| isoquinoline-6-methoxy (CH3) | Phe247 | vdW | 3.4 |

| **4XRZ** | | | |
|---|---|---|---|
| **ligand** | **enzyme-AA** | **mode of interaction** | **dDistance Å** |
| benzene ring | **Phe120** | vdW | 4.0 |
| hydroxyphenyl-OH | Gln244 | H-bond | 2.8 |
| protonated N | **Ser304** | H-bond | 4.6 |
| isoquinoline-7-OH | **Glu216** | H-bond | 3.5 |

**Supplementary Table S9. Compound 9 was docked in three different PDB structures. The inhibitor-enzyme interactions are listed below and the consensus interactions are highlighted in bold.**

| **4WNT** | | | |
|---|---|---|---|
| **ligand** | **enzyme-AA** | **mode of interaction** | **distance Å** |
| benzene ring | **Phe120** | vdW | 3.8 |
| protonated N | **Glu216** | ionic | 5.1 |
| | Asp301 | ionic | 3.3 |
| isoquinoline-7-OH | **Gln244** | H-bond | 3.6 |
| | Ser304 | H-bond | 4.6 |

| **4WNU** | | | |
|---|---|---|---|
| **ligand** | **enzyme-AA** | **mode of interaction** | **distance Å** |
| benzene ring | **Phe120** | vdW | 4.6 |
| methoxyphenyl-O | **Glu216** | H-bond | 3.7 |
| hydroxyphenyl-OH | **Glu216** | H-bond | 3.1 |
| protonated N | Asp301 | ionic | 3.6 |
| isoquinoline-7-OH | **Gln244** | H-bond | 3.4 |
| isoquinoline-6-methoxy (CH3) | Phe247 | vdW | 3.6 |

| **4XRZ** | | | |
|---|---|---|---|
| **ligand** | **enzyme-AA** | **mode of interaction** | **distance Å** |
| benzene ring | **Phe120** | vdW | 3.9 |
| methoxyphenyl-O | **Gln244** | H-bond | 3.6 |
| hydroxyphenyl-OH | **Gln244** | H-bond | 3.2 |
| protonated N | Ser304 | H-bond | 3.7 |
| isoquinoline-7-OH | **Glu216** | H-bond | 3.4 |
| isoquinoline-6-methoxy (O) | **Glu216** | H-bond | 4.4 |

## CypRules performance comparison

The direct comparison of the pharmacophore model performance with CypRules is actually rather challenging. The data set used for comparison needs to be unknown prior to both of the classifiers, because literature datasets , e.g. PubChem, will certainly contain inhibitors that have been used for training the models and thus are already known. The only data set for which this novelty was granted was our own test compound set from this study.

Both the active and the inactive compounds (Table 2 in the main manuscript) were tested on the freely accessible CypRules platform, which is a rule-based P450 inhibition prediction server (http://cyprules.cmdm.tw)[21]. For the calculations, those compounds were classified as active, which exhibited CYP2D6 $IC_{50}$ values up to 10 µM, whereas inactives showed less than 50 % total luminescence at a concentration of 100 µM. The rule-based CYP2D6 inhibition prediction server correctly identified eight out of the thirteen actives as inhibitors. In the *in vitro* screening, the misclassified five actives *i.e.* **15**, **7**, **18**, **8** and **6** exhibited $IC_{50}$ values of 197.8, 582.2, 2109, 4601 and 7022 nM, respectively. Looking at the six compounds that did not show CYP2D6 inhibition *in vitro*, the CypRules platform correctly identified five as inactives *i.e* **4**, **12**, **13**, **16** and **2**. Compound **10** was found to be an inhibitor, although being inactive in the *in vitro* pre-screen assay (Supplementary Table S10). In summary, CypRules correctly classified 62 % of the active and 83 % of the inactive compounds tested in this study.

**Supplementary Table S10. The active compounds were tested on the freely accessible CypRules platform in order to predict their CYP2D6 inhibition.**

| $IC_{50s}$ [nM] | c~~C~~ompounds | CypRules |
|---|---|---|
| 22.45 | **21** | inhibitor |
| 54.70 | **19** | inhibitor |
| 63.64 | **9** | inhibitor |
| 116.9 | **14** | inhibitor |
| 197.8 | **15** | no inhibitor |
| 380.1 | **11** | inhibitor |
| 418.6 | **22** | inhibitor |
| 582.2 | **7** | no inhibitor |
| 2109 | **18** | no inhibitor |
| 4601 | **8** | no inhibitor |
| 7022 | **6** | no inhibitor |
| 8157 | **17** | inhibitor |
| 8885 | **3** | inhibitor |
| inactives | **4** | non inhbibitor |
| inactives | **10** | inhibitor |
| inactives | **12** | non inhbibitor |
| inactives | **13** | non inhbibitor |
| inactives | **16** | non inhbibitor |
| inactives | **23** | non inhbibitor |

A direct comparison of the performance with the approach presented in this manuscript is difficult because only one of the compounds that was predicted as inactive by the model (compound **24**) was part of our test-set. Thus an assessment of the prospective model performance regarding compounds that are predicted to be inactive is not possible. However, the screening of the PubChem BioAssay database showed that our model successfully classified 96 % of the inactive compounds correctly.

## References


1 Schuster, D., Laggner, C., Steindl, T. M. & Langer, T. Development and validation of an in silico P450 profiler based on pharmacophore models. *Current drug discovery technologies* **3**, 1-48 (2006).

2 Usia, T., Watabe, T., Kadota, S. & Tezuka, Y. Cytochrome P450 2D6 (CYP2D6) inhibitory constituents of Catharanthus roseus. *Biological & pharmaceutical bulletin* **28**, 1021-1024 (2005).

3 Li, J. *et al.* In vitro metabolic interactions between black cohosh (Cimicifuga racemosa) and tamoxifen via inhibition of cytochromes P450 2D6 and 3A4. *Xenobiotica; the fate of foreign compounds in biological systems* **12**, 1021-1030 (2011).

4 VandenBrink, B. M., Foti, R. S., Rock, D. A., Wienkers, L. C. & Wahlstrom, J. L. Prediction of CYP2D6 drug interactions from in vitro data: evidence for substrate-dependent inhibition. *Drug metabolism and disposition: the biological fate of chemicals* **40**, 47-53 (2012).

5 Zhao, Y., Hellum, B. H., Liang, A. & Nilsen, O. G. The in vitro inhibition of human CYP1A2, CYP2D6 and CYP3A4 by tetrahydropalmatine, neferine and berberine. *Phytotherapy research : PTR* **26**, 277-283 (2012).

6 Salminen, K. A. *et al.* Inhibition of human drug metabolizing cytochrome P450 enzymes by plant isoquinoline alkaloids. *Phytomedicine* **18**, 533-538 (2011).

7 Hayhurst, G. P. *et al.* Influence of phenylalanine-481 substitutions on the catalytic activity of cytochrome P450 2D6. *The Biochemical journal* **355**, 373-379 (2001).

8 Ji, H. Y. *et al.* Corydaline inhibits multiple cytochrome P450 and UDP-glucuronosyltransferase enzyme activities in human liver microsomes. *Molecules (Basel, Switzerland)* **16**, 6591-6602 (2011).

9 Vrba, J. *et al.* Metabolism of palmatine by human hepatocytes and recombinant cytochromes P450. *Journal of pharmaceutical and biomedical analysis* **102**, 193-198 (2015).

10 Han, Y. L. *et al.* In vitro inhibition of Huanglian [Rhizoma coptidis (L.)] and its six active alkaloids on six cytochrome P450 isoforms in human liver microsomes. *Phytotherapy research : PTR* **25**, 1660-1665 (2011).

11 Yao, Y. M. *et al.* Effect of sinomenine on human cytochrome P450 activity. *Clinica chimica acta; international journal of clinical chemistry* **379**, 113-118 (2007).

12 Mukkavilli, R. *et al.* Noscapine recirculates enterohepatically and induces self-clearance. *European journal of pharmaceutical sciences : official journal of the European Federation for Pharmaceutical Sciences* **77**, 90-99 (2015).

13 Ingelman-Sundberg, M. Genetic polymorphisms of cytochrome P450 2D6 (CYP2D6): clinical consequences, evolutionary aspects and functional diversity. *The pharmacogenomics journal* **5**, 6-13 (2005).

14 Sturm, S., Seger, C. & Stuppner, H. Analysis of Central European Corydalis species by nonaqueous capillary electrophoresis-electrospray ion trap mass spectrometry. *Journal of chromatography. A* **1159**, 42-50 (2007).

15 Choomuenwai, V. *et al.* The discovery, synthesis and antimalarial evaluation of natural product-based polyamine alkaloids. *Tetrahedron Letters* **54**, 5188-5191 (2013).

16 Khokhar, S. *et al.* Isolation, structure determination and cytotoxicity studies of tryptophan alkaloids from an Australian marine sponge Hyrtios sp. *Bioorganic & Medicinal Chemistry Letters* **24**, 3329-3332 (2014).

17 Jones, G., Willett, P., Glen, R. C., Leach, A. R. & Taylor, R. Development and validation of a genetic algorithm for flexible docking. *Journal of molecular biology* **267**, 727-748 (1997).

18 The Cambridge Crystallographic Data Center (2013). GOLD version 5.2, C., Cambridge, UK, https://www.ccdc.cam.ac.uk/ & solutions/csd-discovery/components/gold/.
19 Wang, A., Stout, C. D., Zhang, Q. & Johnson, E. F. Contributions of ionic interactions and protein dynamics to cytochrome P450 2D6 (CYP2D6) substrate and inhibitor binding. *The Journal of biological chemistry* **290**, 5092-5104 (2015).
20 Brodney, M. A. *et al.* Utilizing structures of CYP2D6 and BACE1 complexes to reduce risk of drug-drug interactions with a novel series of centrally efficacious BACE1 inhibitors. *Journal of medicinal chemistry* **58**, 3223-3252 (2015).
21 Shao, C. Y. *et al.* CypRules: a rule-based P450 inhibition prediction server. *Bioinformatics (Oxford, England)* **31**, 1869-1871 (2015).
